# Evaluation of telephone first approach to demand management in English general practice: observational study

**DOI:** 10.1136/bmj.j4197

**Published:** 2017-09-28

**Authors:** Jennifer Newbould, Gary Abel, Sarah Ball, Jennie Corbett, Marc Elliott, Josephine Exley, Adam Martin, Catherine Saunders, Edward Wilson, Eleanor Winpenny, Miaoqing Yang, Martin Roland

**Affiliations:** 1 Cambridge Centre for Health Services Research, RAND Europe, Westbrook Centre, Cambridge CB4 1YG, UK; 2 University of Exeter Medical School, Smeall Building, St Luke’s Campus, Exeter EX1 2LU, UK; 3 RAND Corporation, 1776 Main Street, Santa Monica, CA 90401-3208, USA; 4 Cambridge Centre for Health Services Research, Institute of Public Health, University of Cambridge, Forvie Site, Cambridge CB2 0SR, UK

## Abstract

**Objective** To evaluate a “telephone first” approach, in which all patients wanting to see a general practitioner (GP) are asked to speak to a GP on the phone before being given an appointment for a face to face consultation.

**Design** Time series and cross sectional analysis of routine healthcare data, data from national surveys, and primary survey data.

**Participants** 147 general practices adopting the telephone first approach compared with a 10% random sample of other practices in England.

**Intervention** Management support for workload planning and introduction of the telephone first approach provided by two commercial companies.

**Main outcome measures** Number of consultations, total time consulting (59 telephone first practices, no controls). Patient experience (GP Patient Survey, telephone first practices plus controls). Use and costs of secondary care (hospital episode statistics, telephone first practices plus controls). The main analysis was intention to treat, with sensitivity analyses restricted to practices thought to be closely following the companies’ protocols.

**Results** After the introduction of the telephone first approach, face to face consultations decreased considerably (adjusted change within practices −38%, 95% confidence interval −45% to −29%; P<0.001). An average practice experienced a 12-fold increase in telephone consultations (1204%, 633% to 2290%; P<0.001). The average duration of both telephone and face to face consultations decreased, but there was an overall increase of 8% in the mean time spent consulting by GPs, albeit with large uncertainty on this estimate (95% confidence interval −1% to 17%; P=0.088). These average workload figures mask wide variation between practices, with some practices experiencing a substantial reduction in workload and others a large increase. Compared with other English practices in the national GP Patient Survey, in practices using the telephone first approach there was a large (20.0 percentage points, 95% confidence interval 18.2 to 21.9; P<0.001) improvement in length of time to be seen. In contrast, other scores on the GP Patient Survey were slightly more negative. Introduction of the telephone first approach was followed by a small (2.0%) increase in hospital admissions (95% confidence interval 1% to 3%; P=0.006), no initial change in emergency department attendance, but a small (2% per year) decrease in the subsequent rate of rise of emergency department attendance (1% to 3%; P=0.005). There was a small net increase in secondary care costs.

**Conclusions** The telephone first approach shows that many problems in general practice can be dealt with over the phone. The approach does not suit all patients or practices and is not a panacea for meeting demand. There was no evidence to support claims that the approach would, on average, save costs or reduce use of secondary care.

## Introduction

Many UK general practices are struggling with rising demand from patients, more work being transferred from secondary to primary care, and increasing difficulty in recruiting general practitioners.^1^ ^2^ Countries other than the UK are also facing issues of matching workforce supply to increasing demand for primary care, including the US^3^ ^4^ and Australia.^5^ The US Institute for Healthcare Improvement (IHI) has suggested a wide range of strategies to try to match capacity with demand, including more use of nurses, email consultations, pharmacy clinics, telephone consulting, and carefully planned scheduling to balance supply with periods of high and low demand.^6^ ^7^

One response that has been adopted by around 150 general practices in England is a “telephone first” approach, a practice-wide change in which every patient asking to see a GP is initially phoned back by a GP on the same day, so that patients are unable to see a GP unless they have spoken to a doctor on the phone first. At the end of this phone call the GP and the patient decide whether the problem needs a face to face consultation (usually on the same day) or whether it has been satisfactorily resolved on the phone. Two commercial companies (advertised as Doctor First and GP Access)^8^ ^9^ provide similar types of management support for practices adopting the new approach, with claims that the approach dramatically reduces the need for face to face consultation, reduces workload stress for GPs and practice staff, increases continuity of care, reduces attendance at emergency departments and emergency hospital admission, and increases patient satisfaction. Both companies advocate a GP led approach in which GPs (rather than nurses) telephone patients to determine if the problem can be resolved satisfactorily on the phone.

Some of these claims are repeated in NHS England literature, including the assertion that practices using the approach have a 20% lower use of emergency departments and that “the model has shown cost savings of about £100 000 (€111 000, $133 000) per practice through prevention of avoidable attendance and admissions to hospital.”^10^ Several clinical commissioning groups have subsequently paid for the management support required for the approach to be adopted by practices in their area. In part because claims of benefit for the telephone first approach are based solely on data from the two commercial companies, the National Institute for Health Research commissioned this independent service evaluation. The evaluation determined the impact for practices using the telephone first approach supported by the two commercial companies on practice workload, satisfaction among patients and staff, continuity of care, emergency department attendance, emergency hospital admission, and the associated costs of the approach.

## Methods

We carried out patient surveys in practices using the telephone first approach and analysed trends in consultations in those practices using appointment systems data. We also compared practices using the telephone first approach with control practices in England using data on patient experience from the national GP Patient Survey and data on use of secondary care from hospital episode statistics. Subject to exclusions, we used the same control practices in all analyses. Full details of the analytical methods used are in appendix 1.

### Use of primary care

One commercial provider (GP Access) provided data from routine practice records of 59 practices, which enabled us to examine changes in number and type of appointment, time between booking an appointment and the appointment taking place, length of consultation (judged from the time the patient record was opened until it was closed), and continuity of care (quantified as the proportion of consultations that were with the GP most commonly seen in that month). We included in the analysis GP appointments only on weekdays (full details of inclusion criteria are given in table A in appendix 1). We used mixed effects regression analysis to investigate step changes within a practice associated with the introduction of the telephone first approach and changes in trends relative to the start date. Regression coefficients for step changes and trends were used to quantify the effect of the intervention. When we used Poisson regression (for numbers of appointments) or log transformed data (total time spent consulting), we exponentiated regression coefficients to give effects in terms of ratios. Wald tests of regression coefficients were used throughout. No control data were available for this before and after analysis.

As the intervention started at different times in each practice, we identified the date when each practice started using the new system; when data were available over a period of time we analysed the data relative to this point (the start date).

### Use of secondary care

We obtained hospital episode statistics data from NHS Digital for April 2008 to March 2016. For each practice operating the telephone first approach, we calculated rates of attendance at emergency departments, emergency admission, elective admission, and outpatient attendance for 12 months before and after the date when the telephone first approach was introduced. To allow for external factors, we compared data from all practices known to be operating the telephone first approach (n=145, both commercial providers) with data from a random 10% sample of practices in England (n=829). We used a mixed effects Poisson regression to identify step changes within a practice around the start date and changes in trends relative to the start date. Regression coefficients have been exponentiated to give effects in terms of rate ratios. Wald tests of regression coefficients are used throughout.

### Patient surveys

We analysed responses to the national GP Patient Survey from July 2011 to April 2016, comparing all practices known to be using the telephone first approach (n=146, both commercial providers) with a random 10% sample of other practices in England (n=842). Survey responses were available from 29 472 patients in telephone first practices during this period. We linearly rescaled responses to patient experience items to give a scale from 0 to 100, as in previous work,^11^ ^12^ to facilitate comparison between items. We used mixed effects linear regression analysis to investigate step changes within a practice associated with adoption of the telephone first approach and changes in trends relative to the start date compared with background trends seen in control practices. Finally, we performed a supplementary analysis to investigate if the effect of the intervention was differential between those in or not in employment by including a main effect for working status (based on GP Patient Survey responses) and an interaction between working status and the intervention variable.

We also sent a questionnaire to 80-100 patients who had telephone contact with a GP in each of 20 practices using the telephone first approach (appendix 2). These were practices (out of 101 that had been operating the telephone first approach for at least six months at the time) that agreed to take part in the patient survey. If there were fewer than 80 patient contacts in the day that was sampled, we extracted data on all patients who had spoken to a GP in the two previous days. If there were more than 100 patients (either in a single day or across multiple days needed to get to a sample size of at least 80), we took a random sample of 100 patients. We posted the survey to adult patients and to the parents of patients aged under 13 with a letter from the practice inviting them to respond (patients aged 13-17 were excluded for reasons of patient confidentiality). A reminder was posted to non-responders two weeks after the original mailing. To reduce non-response bias we weighted the results based on the age and sex of the patients who were sent a survey. The survey asked about the experience of the telephone first approach and whether the patient preferred it to the previous system, as well as basic demographic information.

### Economic analysis

In the economic analysis, we estimated primary care costs of introducing the telephone first approach and changes to costs of secondary care and prescribed medicines in practices known to be using the telephone first approach compared with a 10% random sample of English practices (829). We analysed data before and after the intervention start date and against pre-intervention trends. Full details of the methods used in the economic analysis are given in appendix 1.

### Intention to treat analysis

For use of primary and secondary care and national GP Patient Survey data, we conducted the main analyses on an intention to treat basis, including all 146 practices identified by the commercial companies, even when the companies informed us that the practices had adopted a hybrid form of triage or were no longer running the system. This was done to avoid selection bias that would occur if we included only successful practices that continued with the system in the recommended form. We also asked the commercial companies which of the practices they believed were operating the new approach fully or largely consistent with their protocols, and, on the basis of their replies, we performed per protocol sensitivity analyses restricted to this subset of practices. The results presented in the paper are those of the intention to treat analysis, with the sensitivity per protocol analyses, which gave broadly similar results, in appendix 3.

We also carried out a survey of practice managers, which is reported in appendix 4.

### Patient involvement

A study steering group was established, which included four patients. The steering group met on three occasions and provided input into the design and conduct of the study including advice on patient materials produced during the study. Patients were not involved in recruiting practices or patients to the study (patients surveyed were those who had recently had a telephone consultation in one of the intervention practices). As we do not have contact details of these patients are unable to share results with them (questionnaires were sent by the practices). Patient representatives from the steering group attended a learning event at which practices shared their experiences of the telephone first approach and commented on our initial findings. Patients from practices using the system also attended this event and contributed to discussions.

## Results

Table 1 shows the characteristics of practices using the telephone first approach.

**Table 1 tbl1:** Characteristics of 147 general practices using telephone first approach

	No (%) of practices
Commercial provider:	
Doctor First	80 (54)
GP Access	67 (46)
Payer:	
Self pay	74 (50)
CCG	63 (43)
Unknown	10 (7)
List size^*^:	
<5000	18 (12)
5000-10 000	54 (37)
>10 000	73 (50)
No of GPs in practice^†^:	
<4	42 (29)
4-7	53 (37)
>7	50 (35)
Rurality^‡^:	
Urban	139 (95)
Rural	8 (5)
Deprivation fifth^§^:	
1 (least deprived)	15 (10)
2	31 (21)
3	40 (27)
4	31 (12)
5 (most deprived)	29 (20)

*As of September 2015. Data from NHS Digital Data unavailable for two practices that were no longer operating at this time.

†Excludes registrars, retainers, and locums

‡Definition Office for National Statistics based on practice postcode.

§Based on national distribution of practice averages of 2015 index of multiple deprivation. Data unavailable for one practice.

### Use of primary care

There was an overall increase in the number of consultations after the introduction of the telephone first approach from a mean of 16.5 (SD 6.3) to 21.8 (SD 8.1) consultations/day/1000 patients. The change was made up of a substantial reduction in face to face consultations, which reduced from a mean of 13.0 (SD 4.5) to 9.3 (SD 5.5), and an increase in telephone consultations from a mean of 3.0 (SD 4.5) to 12.2 (SD 7.5) telephone consultations/day/1000 patients. Figures 1 and 2[Fig f1 f2] show these results.

**Figure f1:**
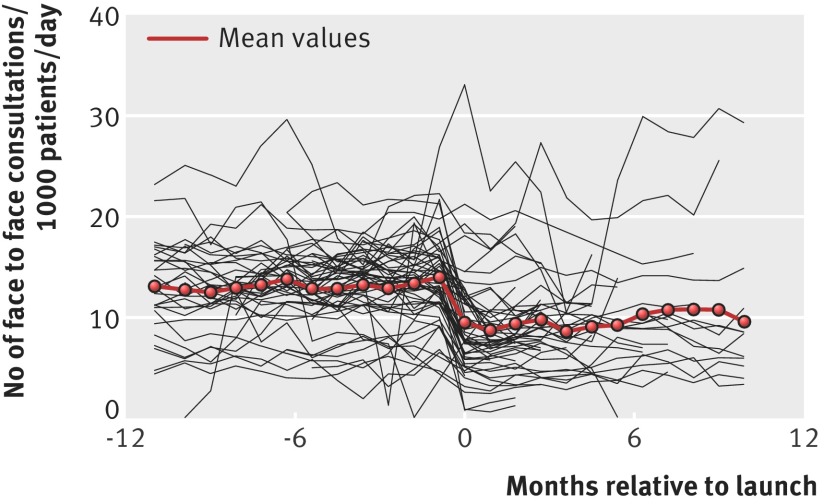
**Fig 1** Change in number of face to face consultations before and after introduction of telephone first approach. Individual lines are crude results from individual practices with mean value

**Figure f2:**
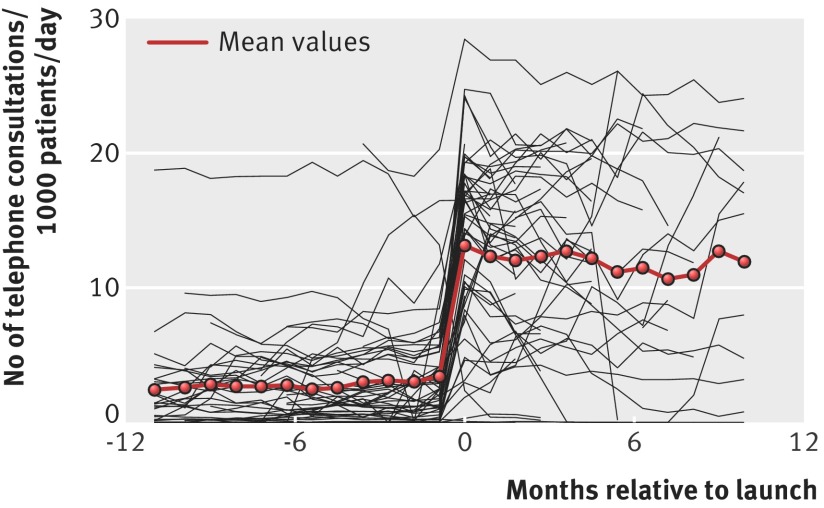
**Fig 2** Change in number of telephone consultations before and after introduction of telephone first approach. Individual lines are crude results from individual practices with mean value

These changes were reflected in the regression results within practices (table 2), which showed a 28% step increase in total consultations and a further increase over the next year. After the introduction of the telephone first approach, face to face consultations decreased considerably (adjusted change within practices −38%, 95% confidence interval −45% to −29%; P<0.001). An average practice experienced a 12-fold increase in telephone consultations (1204%, 633% to 2290%; P<0.001). These average workload figures mask wide variation between practices, with some practices experiencing a substantial reduction in workload and others a large increase. Disparities between the regression results and crude average change reflect the fact that regression results relate to changes within practices, and when there is an association between pre-intervention level and change, the average of changes might not reflect the overall difference. This is particularly noticeable for telephone consultations, where the increase is likely to be smaller for those already performing large number of telephone consultations. Including changes in the number and duration of face to face and telephone consultations, we estimated an overall increase of 8% in the mean time spent consulting by GPs, although with large uncertainty on this estimate (95% confidence interval −1% to 17%; P<0.09). There was a small increase in continuity of care index after the introduction of the new system, though with some decline over the following years. Figures illustrating these changes (superposed epoch analyses similar to figures 1 and 2[Fig f1 f2], which show changes for each individual practice as well as averages) and further details of regression results are shown in appendix 3. Per protocol analyses of the subset of practices operating the telephone first approach in line with the companies’ protocols throughout showed broadly similar results and are also shown in appendix 3. It is important to note that all the average figures mask wide variation between practices, as indicated by the model random effects. This ranged, for example, from practices experiencing a doubling in the total number of consultations to others in which there was overall net reduction in workload, with face to face consultations reduced by over two thirds (table 2).

**Table 2 tbl2:** Results of mixed effects regressions showing within practices of intervention on use of primary care*

Outcome	Step change at transition				Pre-transition trend			Post-transition trend		Interaction P value‡
	Effect size (95% CI)	P value	Heterogeneity†		Effect size per year per year (95% CI)	P value		Effect size per year (95% CI)	P value	
Change in consultations numbers (rate ratio)										
Total No of consultations§	1.28 (1.17 to 1.39)	<0.001	0.68-2.39		1.07 (1.06 to 1.08)	<0.001		1.04 (1.04 to 1.05)	<0.001	<0.001
No of face to face consultations§	0.62 (0.55 to 0.71)	<0.001	0.24-1.62		1.03 (1.01 to 1.04)	<0.001		0.98 (0.97 to 0.99)	<0.001	<0.001
No of telephone consultations§	12.04 (6.33 to 22.90)	<0.001	0.10-1467.39		1.11 (1.09 to 1.12)	<0.001		1.46 (1.43 to 1.49)	<0.001	<0.001
Change in total time spent consulting (ratio)										
Total time spent consulting¶	1.08 (0.99 to 1.17)	0.088	0.65-1.79		1.00 (0.94 to 1.05)	0.87		1.05 (1.02 to 1.09)	0.005	0.0856
Change in length of consultation (difference in minutes)										
Length of consultations (all types)**	−0.88 (−1.43 to −0.33)	0.002	−3.67-1.91		0.19 (0.14 to 0.25)	<0.001		−0.28 (−0.35 to −0.21)	<0.001	<0.001
Length of face to face consultations**	0.22 (−0.11 to 0.55)	0.18	−1.41-1.86		0.35 (0.29 to 0.41)	<0.001		−0.34 (−0.43 to −0.24)	<0.001	<0.001
Length of telephone consultations††	−0.51 (−0.89 to −0.13)	0.010	−1.79-0.77		0.42 (0.28 to 0.57)	<0.001		−0.39 (−0.50 to −0.29)	<0.001	<0.001
Change in continuity index (difference)										
Continuity of care**	0.058 (0.037 to 0.081)	<0.001	−0.074-0.191		−0.0001 (−0.0002 to 0.0001)	0.40		−0.006 (−0.006 to −0.005)	<0.001	<0.001

*Adjusted for month and day of week and random intercept for practice to account for different baseline level. Duration of data included in analyses varies by practice and by outcome but was maximum of 12 months before and 12 months after change in each practice: random effect analysis means that models are not biased by missing data.

†In terms of 95% mid-range for practices—range of rate ratios for “true” step changes expected 95% of practices after adjustment for patient sample size. Estimated with SD of random slope for step change (*σ_step_*) combined with fixed effect of step change (*β_step_*) as e*^βstep^* ^±1.96^ *^σstep^* or *β_step_*±1.96*σ_step_* for ratios and differences, respectively.

‡Interaction P value is for test of whether post-transition trend is different to pre-transition trend.

§Mixed effects Poisson regression.

¶Mixed effects linear regression on log transformed data. Exponentiated coefficients given to provide duration ratios.

**Mixed effects linear regression.

### Patient surveys

Table 3 compares the responses to the national GP Patient Survey from practices using the telephone first approach and other practices in England. There was a large and immediate improvement in patients’ rating of time to be seen or spoken to (increase of 20 points on a 0-100 point scale) compared with national trends. All other differences were small in comparison and included small negative effects on scores for communication with the GP, ability to see the patient’s preferred GP, and willingness to recommend the surgery to others. The per protocol analysis carried out for the subset of practices operating the telephone first approach in line with the companies’ protocols throughout showed similar results (appendix 3). As with the use of primary care data, these average figures mask large variation between practices (as evidenced by random effects) except in the rating of time to be seen or spoken to, when all practices showed improvement. The supplementary analysis found no evidence that the effect of the intervention was differential between those patients in or not in work (P>0.1 for all, results not shown).

**Table 3 tbl3:** Responses to national GP Patient Survey: comparison between telephone first practices and random samples of other practices in England (variables scored on 0-100 scale, positive number indicates improvement)

Survey outcome	Step change after intervention				Additional yearly change after intervention		
	Difference (95% CI)	P value	Heterogeneity (95% mid-range)^*^		Difference (95% CI)	P value	Heterogeneity (95% mid-range)^*^
GP communication composite	−0.89 (−1.40 to −0.38)	<0.001	−3.83-2.05		−0.03 (−0.29 to 0.23)	0.82	−0.52-0.46
Ease of getting through on phone	0.49 (−0.58 to 1.57)	0.37	−9.07-10.05		0.18 (−0.57 to 0.93)	0.64	−5.82-6.18
Would you recommend your GP surgery	−2.37 (−3.22 to −1.52)	<0.001	−9.11-4.37		0.24 (−0.24 to 0.72)	0.34	−2.54-3.02
Seeing preferred GP	−1.25 (−2.41 to −0.08)	0.035	−7.78-5.28		0.050 (−0.65 to 0.75)	0.89	−3.24-3.34
Time until seen or spoken to	20.04 (18.16 to 21.93)	<0.001	1.44-38.64		0.12 (−0.87 to 1.11)	0.81	−7.62-7.86
Convenience of appointment	0.38 (−0.35 to 1.10)	0.31	−5.11-5.87		0.41 (0.08 to 0.75)	0.016	−0.84-1.66
Overall experience of making appointment	−0.44 (−1.46 to 0.57)	0.39	−9.73-8.85		0.86 (0.32 to 1.40)	0.002	−2.65-4.37

*In terms of 95% mid-range for practices—range for “true” step changes/additional yearly changes expected across 95% of practices after adjustment for patient sample size. Estimated with SD of random slope for step change (*σ_step_*) combined with fixed effect of step change (*β_step_*) as *β_step_*±1.96*σ_step_*

We sent the patient questionnaire we developed (appendix 2) to 1873 patients from 20 practices using the telephone first approach. We received 837 responses, a 44.7% response rate. Table 4 shows the results. Nearly two thirds of patients (weighted percentage 64.9%) reported being called back within an hour. In half of the calls (weighted percentage 50.6%) the patient was asked to come into the surgery for a face to face consultation with a GP or nurse. Advice only was given in 17.3% of phone calls (weighted percentage 16.8%), and 21.9% of phone calls resulted in a prescription being given (weighted percentage 23.9%). In response to the question “Did you find the telephone appointment more or less convenient than just attending a face to face appointment, without being able to talk to the doctor on the phone first?” over half said that the telephone first approach was more convenient with similar percentages reporting that it was less convenient or made no difference. Just under a quarter reported that it was more difficult to communicate with the GP on the phone, the main reasons being given that it was difficult to explain the problem or that the doctor could not see the problem, but nearly two thirds found it made no difference, and 11.7% found it easier to communicate on the phone. Respondents were fairly equally divided over whether they preferred the new system, would prefer to return the old system, or didn’t mind.

**Table 4 tbl4:** Responses to patient survey (837 patients who had received phone call in telephone first practice)

	No (%) of responses	Weighted %
How long did it take for a GP to call you back?		
<20 minutes	189 (23)	21
20-60 minutes	361 (44)	44
>1 hour	256 (31)	34
GP did not call back	9 (1)	1
What was the outcome of the telephone call?		
I received telephone advice only	145 (17)	17
I was given a prescription	183 (22)	24
An appointment with a GP in the surgery	367 (44)	44
An appointment with a nurse in the surgery	61 (7.)	7
A follow-up telephone appointment with a GP	30 (4)	4
A follow-up telephone appointment with a nurse	17 (2)	2
Other	67 (8)	9
Did you find the telephone appointment more or less convenient than just attending a face to face appointment?		
More convenient	426 (55)	56
Less convenient	166 (22)	22
No difference	177 (23)	22
Do you find it more or less difficult to communicate with the GP over the phone than in person?		
More difficult	182 (23)	24
Less difficult	91 (12)	12
No difference	505 (65)	64
If you answered “more difficult” to the previous question, why do you think it was more difficult to communicate over the phone?		
English is not my first language	0	0
The doctor really needs to see me	52 (29)	28
The telephone line was not clear	15 (8)	8
I have impaired hearing	5 (3)	2
I found it difficult to explain the problem	85 (47)	50
Other	4 (2)	2
Would you like to go back to the old system, where most GP appointments were face to face?		
Yes	267 (33)	30
No	250 (31)	32
Don’t know	299 (37)	38

To get agreement from 20 practices to take part in this survey, we had to approach all 101 practices that had been operating the telephone first approach for at least six months at the time of recruitment. Because these might be practices that were operating the new system more successfully, we compared practices that agreed and declined to take part in our survey using national GP Patient Survey data, and confirmed that patient experience was more positive in practices that agreed to take part in our survey (based on GP Patient Survey data, 86% of patients rated the overall experience of making an appointment as good or very good in practices that agreed to take part in the survey compared with 80% in practices that declined). The results of our survey (but not the comparison with the national GP Patient Survey) might therefore be a positively biased representation of patients’ experiences.

Patients had the opportunity to provide free text comments at the end of the questionnaire. Box 1 provides examples of quotations, which illustrate the wide range of views expressed about the new approach. These mirror the range of strongly held positive and negative views that were also found in qualitative interviews with patients (not reported in this paper).

**Box 1: Examples of positive and negative views about the telephone first approach from free text comments included in the patient survey**

**Examples of positive comments**

I think to be able to pick up a phone and speak to your GP, who will either see you, leave a prescription, or advise you to see a nurse, is excellent (practice 104)

On the rare occasions it’s been an emergency, the ring back has been almost immediate—I cannot fault the system (practice 105)

It saves wasting the time of the GP if the matter can speedily be dealt with over the phone (practice 116)

Excellent service. It’s much easier to speak to doctor and the few times I’ve phoned in, my problem has been sorted out over the phone which saves time for both parties (practice 117)

With this system, if it’s necessary, you usually get to see the doctor the same day—it saves time for serious cases to see the doctor. Much better than the old system (practice 108)

**Examples of negative comments**

Speaking to the doctor isn’t the problem: it’s getting through on the phone that’s the difficulty (practice 111)

I think this system is stupid. Once I waited six hours for a call back—I could have been dead (practice 110)

The call back system is truly awful—I cannot plan my day as I work from site to site over long distances (practice 103)

If you work in a job where you can’t take phone calls it can be almost impossible to get a call back from a doctor (practice 121)

The only difficulty for me is that it’s sometimes difficult to explain more intimate matters to a GP over the phone—I work in an office with other people and it can be difficult to find a quiet confidential place to talk (practice 116)

### Use of secondary care

Table 5 shows the changes in use of secondary care within practices. When practices changed to the telephone first approach, there was no change in attendance at emergency departments and a small (2%) increase in emergency admissions, an increase that was greater (4%) for admissions for ambulatory care sensitive conditions (conditions for which admissions could, in principle, be avoided by good primary care). In the year after the change to the new system, there were small (2%) reductions in the previous rate of increase in emergency department attendance and outpatient attendance, compared with national trends, but an acceleration in the previous rate of increase for admissions for ambulatory care sensitive conditions. Heterogeneity identified with a random effect shows that these figures mask considerable differences between individual practices, some of which had large increases in emergency department attendances, with others showing large decreases. The per protocol analysis carried out for the subset of practices operating the telephone first approach in line with the companies’ protocols throughout showed similar results (appendix 3).

**Table 5 tbl5:** Changes in use of secondary care after adoption of telephone first approach in general practice

Outcome	Step change at transition^*^				Additional yearly change after intervention^*^	
	Rate ratio (95% CI)	P value	Heterogeneity^†^		Rate ratio per year (95% CI)	P value
Emergency department attendances	1.00 (0.99 to 1.02)	0.68	0.92-1.10		0.98 (0.97 to 0.99)	0.005
Outpatient attendances	1.00 (0.99 to 1.02)	0.63	0.89-1.13		0.98 (0.97 to 0.98)	<0.001
All admissions	1.02 (1.01 to 1.03)	0.006	0.98-1.05		1.01 (1.00 to 1.02)	0.2
Admissions for ACSCs	1.04 (1.00 to 1.08)	0.032	0.87-1.24		1.06 (1.02 to 1.11)	0.007
Elective admissions	1.01 (0.99 to 1.02)	0.56	0.90–1.13		1.02 (1.00 to 1.04)	0.015
Emergency admissions	1.02 (1.00 to 1.04)	0.016	0.96-1.09		1.00 (0.98 to 1.03)	0.86

ACSCs=ambulatory care sensitive conditions (conditions for which admissions could, in principle, be avoided by good primary care).^13^

*Results of controlled mixed effect Poisson regressions modelling, adjusted for patient demographics, national, seasonal, and long term trend effects, clustering by practice including heterogeneity in baseline scores and trends.

†In terms of 95% mid-range for practices—range of rate ratios for “true” step changes expected across 95% of practices after adjustment for patient sample size. Estimated with SD of random slope for step change (*σ_step_*) combined with fixed effect of step change (*β_step_*) as e*^βstep^* ^±1.96^ *^σstep^*.

### Economic analysis

Eighteen practices provided detailed information on costs of adopting the telephone first approach. The median amount paid to commercial companies for supporting the change was £10 810 per practice (range £7200 to £13 803). In some cases this had been paid by the Clinical Commissioning Group (CCG) rather than by the practice itself. Several practices installed additional phone lines and/or bought new mobile phones. Some practices reported substantial initial increases in the costs of calls, though this could have been a short term issue as more suitable contracts were negotiated. No practices purchased new computer hardware or software as existing systems were able to implement the new approach. Six practices incurred costs related to informing patients about the new system, including updating the practice website, newspaper advertisements, and leaflets. When these were done in house this did not incur any additional cost other than the opportunity cost of administrator time. One practice mailed all their patients with information about the new system at a cost of £7600 (covered by the CCG). Five practices employed additional staff after the change, though they said it was hard to attribute this to the adoption of the telephone first approach. Two practices reported that they had recruited staff because of an increased list size, which they attributed to patients switching from other practices because of easier access to GPs in their practices. One of the 18 practices interviewed appointed an additional full time nurse because of a perceived increase in consultations, though they subsequently decided that the additional nurse was excess to requirements. The introduction of the telephone first approach was not associated with differences in cost or numbers of prescriptions issued except for some minor changes which we judged unlikely to be of clinical importance (see details in tables M and N in appendix 3).

Table 6 shows the change in costs of use of secondary care. When we combined the step change at the introduction of the telephone first approach with the change in underlying trend over the subsequent year, we found small non-significant reductions in costs for emergency department and outpatient attendance and a significant increase in costs of admissions, leading to an estimated overall increase in secondary care costs of £11 766 per 10 000 patients in the first year after the change to the new approach (aggregating emergency department and outpatient attendance (−£577, −£2762) and admissions coded as emergency or elective (+£7993, +£7112)).

**Table 6 tbl6:** Changes in costs of secondary care (£) per 1000 registered patients in general practices that changed to telephone first

	Mean (95% CI) costs				Mean (95% CI) change in cost attributable to telephone first approach^*^		
	Over 12 months before transition	Over 12 months after transition	Crude change		Initial	Over next 12 months	Total change over first 12 months
Emergency department attendance	57 546 (54 948 to 60 144)	59 555 (56 847 to 62 264)	2009 (1074 to 2944)		2 (−853 to 866)	−578 (−870 to −287)^†^	−577 (−1481 to 335)
Outpatient attendance	275 673 (264 037 to 287 309)	293 408 (280 283 to 306 534)	17 735 (12 868 to 22 602)		8 (−4086 to 4148)	−2770 (−3483 to −2064)^†^	−2762 (−6921 to 1434)
Inpatient admissions for ACSCs	99 821 (94 340 to 105 302)	104 997 (99 109 to 110 885)	5176 (1851 to 8500)		4013 (73 to 8083)^†^	2957 (800 to 5160)^†^	6970 (2464 to 11 600)^†^
Inpatient admissions coded as “elective”	399 822 (384 057 to 415 587)	421 051 (403 406 to 438 695)	21 228 (13 437 to 29 019)		4009 (−1987 to 10 077)	3984 (39 to 7966)^†^	7993 (807 to 15 249)^†^
Inpatient admissions coded as “emergency”	354 384 (335 309 to 373 459)	350 183 (331 767 to 368 598)	−4201 (−12 739 to 4337)		7105 (66 to 14 272)^†^	7 (−4385 to 4439)	7112 (−1192 to 15 531)

ACSCs=ambulatory care sensitive conditions (conditions for which admissions could, in principle, be avoided by good primary care).^13^

*Attributable change takes into account background trends in 10% sample of control practices in England.

†Significant.

## Discussion

Our study shows that adoption of the telephone first approach in general practice had a major effect on patterns of consultation, with large increases in phone consultations and decreases in face to face consultations. Our patient survey suggests that up to half of patients’ problems could be dealt with on the phone, which could offer potential for practices struggling with demand for face to face consultations. The telephone first approach, however, is not a panacea for management of demand and is on average associated with increased overall GP workload. Consistent with previous work on telephone triage,^14^ there was an average increase in overall time spent consulting, though our uncertainty on this estimate is large. We also note wide variation between practices ranging from large reductions in GP workload to large increases. The introduction of the telephone first approach is associated with dramatic improvements in patients’ perception of the time to be seen or spoken to but a quarter of patients found it more difficult to communicate with their GP on the phone. Nevertheless, more than half of patients surveyed said they preferred the telephone first approach to their practice’s previous arrangement.

We found no evidence that the approach substantially reduced overall attendance at emergency departments or emergency hospital admissions and some evidence that overall costs of secondary care increased slightly. While we found evidence here and in qualitative results not reported in this paper that some working patients disliked the approach (for example, if they could not take calls at work), there was no net difference in the survey responses of working and non-working patients to new approach. This probably indicates that other working patients found it more convenient (for example, because they no longer needed to take time off work for a consultation with their GP). Although we found evidence of wide variation in the impact of the telephone first approach in different practices, we were not able to fully explain why, for some practices, the approach had transformed their ability to cope with patient demand whereas for others the approach seemed to open floodgates of demand that at times overwhelmed the practice. Supported by informal observations in practices not reported here, our impression is that the approach worked better in highly organised data driven practices that already had a handle on demand and was less likely to prove successful in practices where the ability to cope with demand was already out of control. It was also important that practices had sufficient telephone lines to meet periods of high demand. The management support provided by the two commercial companies enabled practices to have a detailed understanding of patterns of demand during the week so that they could anticipate periods of high demand and allocate resources appropriately, though this seemed to have been implemented more successfully in some practices than others. It was also clear that, though some practices had organised the new system to improve continuity of care, in others patients found it more difficult to see or speak to the doctor of their choice.

### Strengths and limitations of this study

We used a combination of quantitative methods, when possible comparing the telephone first approach with background trends in control practices in England. Strengths of the study include the multi-method approach and the inclusion of all practices that had been using the telephone first approach for at least six months at the start of the study. Among the limitations is the likelihood that practices operating the telephone first approach successfully were more likely to participate in our patient survey and surveys with practice managers, and we found that practices that participated in our survey had, on average, better national patient survey scores than those that were operating the telephone first approach but did not participate. It is possible that the telephone first approach also added to nursing workload in the practices, though we did not measure this. Our economic analysis, however, provided no evidence that practices needed to increase nurse staffing levels as a result of introducing the new system, and in the ESTEEM study^15^ increases in nursing workload were much greater when nurses, rather than GPs, took triage calls (as in this study). We did not include home visits in the analysis as the small numbers made analyses difficult. Some hard to reach groups of patients, including those with poor English, might also have been under-represented in the patient survey responses, and these might be patients who had particular problems with the telephone first approach. In addition, we relied on workload data from practice records that were not originally collected for research purposes. In particular, there was a substantial amount of missing data for consultation length, for which we used imputation when calculating the total time spent consulting, and the corresponding results should be treated with caution. The study also provides no information on health outcomes.

### Comparison with other studies

While most UK practices offer some facility for telephone consultations,^16^ previous research has focused on a triage approach where, for example, requests for on the day consultations or home visits are triaged on the phone by a doctor or nurse. While the impact of these depends on the particular approach being adopted,^17^ and while triage might be an appropriate way of prioritising work within a general practice, it has not consistently been associated with reductions in practice workload.^18^ ^19^ For example, a large randomised trial of GP and nurse led triage for patients seeking same day consultations (ESTEEM) found that it was associated with an increase in the number of primary care contacts over the subsequent 28 days and no evidence of reduction in NHS costs.^15^ ^20^ Likewise, in the US, provision of telephone access for patients increases the availability of medical advice for patients but does not decrease cost, with most telephone consultations in “telehealth” services representing new use.^21^ While the telephone first approach that we evaluated offers a more radical approach to management of demand, the overall conclusion about effect on workload is similar. Concerns have also been expressed that increased use of telephone consulting could compromise patient safety.^22^ Although we have not directly examined patient safety in this paper, staff and patients more often expressed a view that safety was improved by being able to deal with patients’ problems without long waits for appointments.

### Conclusions

For clinicians, this study provides clear evidence that a considerable part of patient workload can be dealt with through phone consultations. This might increase the practice’s control over day to day workload but does not necessarily decrease the need for GP time and could increase it. We found no evidence to support claims reproduced in NHS England literature^5^ that the approach would be substantially cost saving or reduce use of secondary care.

The pure form of the telephone first approach advocated by the commercial companies requires that all face to face consultations are preceded by a phone call (that is, no advance booking is allowed). Though some practices adopted this approach, many had modified the system to allow a degree of advance booking. Unanswered questions remain about the balance between meeting the needs of patients for different types of appointment and a system that allows practices to keep tight control on patients’ requests for face to face consultations. Consistent with our qualitative work (not reported here), patients expressed strong views for and against the new system, and better understanding is needed of the requirements of practices that make the difference between the approach becoming described as excellent or awful. Non-commercial guidance is available to practices that are considering increasing telephone consultations on the issues that need to be considered.^23^

**What is already known on this topic**

GPs are struggling with the current demands on general practice and looking for effective ways to manage patient demand
Claims have been made, reproduced in NHS England literature, that a telephone first approach, in which all patients wanting to see a GP are asked to speak to a GP on the phone first, results in major cost savings for primary care and reductions in secondary care costs

**What this study adds**

In general practice, many problems can be dealt with by a GP on the phone
The new telephone first approach resulted in more phone calls, fewer face to face consultations, and, on average, more time spent consulting
There was wide variation between individual practices, including large increases and large decreases in workload after adoption of the telephone first approach
There was no evidence that the telephone first approach would reduce costs of secondary care

## Web Extra

Extra material supplied by the author

Appendix 1: Further details of analytical methods

Appendix 2: Patient questionnaire

Appendix 3: Further details of results

Appendix 4: Practice manager survey

## Notes

We thank members of our study steering group, including the patient members, who provided advice during the course of the study; the patients, GPs, and the practice manager who were part of our steering group, those who came and contributed to our learning events, and patients who completed questionnaires as part of our patient survey; and the commercial companies (Productive Primary Care and GP Access) that provided contact details of practices using their approaches and the staff and patients from the practices taking part in the study.

Ethical approval: The study was approved by the West of Scotland NHS Research Ethics Service (7th May 2015, REC reference 16/WS/0088).

Data sharing: No additional data are available.

Transparency The lead author affirms that the manuscript is an honest, accurate, and transparent account of the study being reported; that no important aspects of the study have been omitted. There are no significant discrepancies from the study as planned.
