# Supplementary material for: Evaluation of telephone first approach to demand management in English general practice: observational study
Source: BMJ. 2017 Sep 28;358:j4197. doi: 10.1136/bmj.j4197 (PMC5615264; doi:10.1136/bmj.j4197)
Supplement: Supplementary file 1 — Appendix 1: Further details of analytical methods [file newj040067.ww1.pdf]

Appendix 1. Further details of analytic methods [posted as supplied by author]

Our evaluation related to 147 practices using the 'telephone first' approach supported by the two commercial companies. Of these 145 practices were included in the analysis of hospital utilisation and costs because two had closed before the final data collection point that we used. 146 practices were used in the analysis of the GP Patient Survey, data not being available for one practice.

1.1 Methods of analysing primary care utilisation data

Data on phone and face to face appointments, and on continuity of care were extracted from the practices computer systems by the commercial company up to 28th October 2016 and transferred to the research team as anonymised data sets for analysis. For individual appointment-level data information was available regarding: the date and time an appointment was booked; the date and time the appointment occurred; the type of appointment (face-to-face, telephone, home visits or administrative); and who the appointment was with (GP, nurse or other). The continuity of care data included the usual provider continuity score and patient age. To preserve anonymity, the commercial company created the usual provider continuity score[1] calculated for each patient who had two or more appointments in any calendar month, as the number of appointments with the GP most frequently seen divided by the total number of appointments in that time period. Patients could not be linked across different months. The company also provided details on the date the 'telephone first' approach was introduced and the current status of the system i.e. whether still running the 'telephone first' system per protocol, running a hybrid system or if the practices had abandoned the approach. Only practices which launched the 'telephone first' approach before the 31st December 2015 were included in the final data set to allow sufficient time for the system to have bedded in (potentially allowing at least 10 months of post intervention data for each practice, though in reality this was often less).

Two types of analysis were carried out for each of the outcomes. The first was a before and after analysis in intervention practices, illustrated by the 'super-posed epoch graphs' in appendix 3.1 where the introduction of the system in each practice is set at time zero. Second, a regression analysis was performed for each outcome looking first for step changes at the time the intervention was introduced and second for a change in the preceding trend (e.g. slowing down of a previous increase). We also model heterogeneity in these changes to examine whether the intervention has a different effect in different practices.

[1] Breslau N, Reeb KG. Continuity of care in a university-based practice. J Med Educ. 1975;50(10):965–9.

Before and after super-posed epoch analyses

Graphical super-posed epoch analyses were performed for the five main outcomes to illustrate the unadjusted change in outcomes before and after the introduction of the intervention. For all outcomes except continuity of care the average of the outcome was calculated over 30-day periods relative to the launch date; with the practice launch date defining time zero for individual practices. For continuity of care the calculation was based on calendar months due to the format of data provided. Given the intervention started at different time points in each practice, different relative time periods include data from different periods of time.

The outcomes, definitions and analytic approach taken are summarised in table A.

Table A Overview of models used and outcomes studied to determine changes associated with the use of the 'telephone first' approach

Outcome	Definition	Inclusion criteria	Model	Unit of analysis
Number of appointments	Total number of appointments (face-to-face, telephone or home visits) with a GP per practice per day	• GP appointments<br>• Face-to-face, phone or home visits<br>• Appointments on Monday to Friday<br>• Appointments a year either side of launch date	Mixed effects Poisson regression	Each day in each practice
Time waited for an appointment	Number of days between booking the appointment (face to face or telephone) to the time they had the appointment with the GP.	• GP appointments<br>• Face-to-face or phone<br>• Appointments on Monday to Friday<br>• Appointments booked Monday to Friday only	Linear mixed model	Individual appointments
Length of appointment	The time in minutes between the start and end of an appointment (face-to-face or telephone) with a GP	• GP appointments<br>• Face-to-face or phone<br>• Appointments on Monday to Friday<br>• Apparently -ve appointment lengths. Appointment lengths of 0 minutes or > 30 minutes excluded.	Linear mixed model	Individual appointments
Total time spent consulting (by GPs) per day	The total time in minutes GPs spent in appointments (face-to-face and telephone) with patients per practice per day.	• GP appointments<br>• Face-to-face or phone<br>• Appointments on Monday to Friday	Log-transformed for analysis.<br>Linear mixed effects regression model	Each day in each practice
Continuity of care	For patients with two or more appointments in a month, the proportion of appointments that are with the GP most frequently seen in that month (score from 0 to 1).	• GP appointments<br>• Face-to-face appointments only	Linear mixed effects regression	Individual patients in each month

For each outcome we calculated: (1) the mean within an individual practice for each time period; and (2) the mean of individual practice means with available data for each time period. These means were plotted as time relative to launch.

Regression analysis

Mixed effects regression analysis was used to investigate within practice changes associated with the intervention. The models used all take a similar form, but different types of model were used depending on the outcome. In brief the models captured a step change associated with the start of the intervention as well as a change in trend, for example the intervention may have led to an immediate increase relative to the background trend which was then eroded by a reduction over time. We also model heterogeneity in these changes to examine whether the intervention has a different effect in different practices. A complete case analysis was used with the exception of the total time spent consulting.

Each model contained a categorical variable for month to account for seasonality and a categorical variable for day of week to account for variations across the week. Continuous time relative to launch date (in years) was included to account for underlying secular trends. A random intercept was included to account for differing baselines by practice and the resulting clustering of observations within practices. A binary variable indicating when the intervention was present captured any instantaneous "step" change in the outcome at the start of the intervention. An interaction between time relative to launch and the intervention indicator captured whether the linear trend changed following intervention. For continuity of care models only, the age of the patient was also included as a third order (cubic) polynomial in addition to the variables described above. Analyses were performed for all types of appointments combined as well as separately by type of appointment (face to face or telephone).

A large amount of data on length of appointment was missing from the practice data provided by the commercial company (30%), especially so for telephone consultations (52% compared with 18% for face to face consultations). Ignoring appointments with missing durations would have led to a systematic underestimation of the total time spent consulting. To overcome this appointment length was imputed for those appointments with missing length and then added to the observed lengths to obtain a better estimate of the total time spent consulting for each day in each practice. Because we were imputing individual consultation lengths rather than total time spent consulting per day in a practice a single imputation was made using a linear regression model similar that used in the analysis of individual consultation lengths but with fixed effects for practice rather than random ones and stratified by before and after intervention launch. As required to avoid biasing standard errors under single imputation, the

imputed standard errors were multiplied by the square root of the ratio of the proportion of cases requiring no imputation.

To better approximate normal distributions, data were log-transformed prior to analysis. For the ease of interpretation exponentiated coefficients are presented as duration ratios (i.e. the relative change in total time spent consulting by a practice).

Our main analysis was done on an intention to treat basis. It includes all practices identified by the commercial company even when the practices were using a hybrid form of the 'telephone first' approach or had since ceased using it all together. A sensitivity analysis (appendix 2) was performed restricting the analysis to practices where we believed, on the basis of information provided by the commercial company, that the system was being run consistent with the company's protocols.

1.2 Methods of analysing national GP Patient Survey data

The GP Patient Survey (GPPS) is a national postal survey of patients' experience of primary care in England. The survey is sent twice a year (July to September and January to March) to around 2.8 million adult patients a year. A stratified sample of patients aged 18 and over who have been continuously registered with a general practice for at least 6 months is drawn from the practice list of each general practice in England, with patients from practices known from prior surveys to have low response rates oversampled. Full details of the sampling strategy are published elsewhere[2].

We used data from July 2011 to April 2016. The contents of the survey have remained largely consistent over this time period. Response rates have fluctuated over the period; they fell from 38% in 2011/12 (1,037,946 responses) to 33% in 2014/15 (858,381 responses) and recovered to 39% in 2015/16 (836,312 responses) following the introduction of a reminder postcard in addition to the two reminder surveys already used. The data period included data from a total of 8,323 practices, but not all practices had data for each wave of the survey; the number of practices contributing data varied between 8,243 and 7,687 in any one wave. This is primarily due to practice closures and openings.

Practices using a 'telephone first' approach between 2011 and 2016 were identified based on information provided by the two commercial companies providing support for implementing this service (hereafter we refer to these practices as intervention practices). The companies provided details on the practices that were using their system, the date the 'telephone first' approach was introduced and the current status of the system i.e. whether still running a full 'telephone first' system, running a hybrid system or if the practices had abandoned the system. Only practices which launched the 'telephone first'

[2] https://gp-patient.co.uk/surveys-and-reports

approach before the 31st December 2015 were included in the analysis to allow sufficient time for the system to have bedded in; those with later launch dates were classified as non-intervention practices. In total 146 intervention practices were identified in the GPPS data set. The number of practices receiving the intervention varied over time (see Table B); only one practice was using the 'telephone first' approach throughout the entire data period. A total of 29,472 surveys were received from intervention practices post launching the 'telephone first' approach.

Table B The number of practices using the 'telephone first' approach in each survey wave

Wave	Year				
	**2011/12**	**2012/13**	**2013/14**	**2014/15**	**2015/16**
Jul/Sep	1	22	74	105	140
Jan/Mar	6	44	79	122	144

We used seven measures of patient experience from the GP Patient Survey in this analysis:

1. Last time you saw or spoke to a GP from your GP surgery, how good was that GP at each of the following?
   - Giving you enough time
   - Listening to you
   - Explaining tests and treatments
   - Involving you in decisions about your care
   - Treating you with care and concern
2. Generally, how easy is it to get through to someone at your GP surgery on the phone?
3. Would you recommend your GP surgery to someone who has just moved to your local area?
4. How often do you see or speak to the GP you prefer?
5. How long after initially contacting the surgery did you actually see or speak to them?
6. How convenient was the appointment you were able to get?
7. Overall, how would you describe your experience of making an appointment?

In each case responses were rescaled between 0 (poor experience) and 100 (good experience). In the case of the first question above on 'GP communication', a composite variable was created taking the mean of all informative responses as long as three or more informative responses were given.

Two types of analysis were carried out for each of the outcomes. The first was a before and after analysis, illustrated by the 'super-posed epoch graphs' where the introduction of the system in each practice is set at time zero. Second, a regression analysis was performed for each outcome looking first for step changes at the time the intervention was introduced and second for a change in the preceding

trend (e.g. slowing down of a previous increase). We also model heterogeneity in these changes to examine whether the intervention has a different effect in different practices.

Before and after analysis of GPPS scores (intervention practices only)

Graphs have been produced illustrating changes in patient experience scores in intervention practices before-and-after the introduction of the intervention (shown in appendix 3). A super-posed epoch analysis is performed whereby the number of survey waves relative to the intervention launch date is calculated for each practice; the survey immediately preceding the intervention launch date is defined as time zero, the survey wave immediately following intervention launch date is time period one, and the one following that time period two etc. Given the intervention started at different time points in each practice, different relative time periods include data from different periods of time. Further not all practices had data for all time periods relative to intervention launch. The analysis was restricted to data two years either side of the launch date.

For each of the seven GPPS experience measures we calculated: (1) the mean score within each intervention practice for each relative time period; and (2) the mean across all intervention practices with available data for each relative time period. These means were plotted as time relative to launch.

Comparison with other practices in England (controlled regression analysis)

The super-posed epoch analysis does not control for what is happening external to the intervention and may also be confounded by a number of factors. For example, the timing of intervention launch relative to nationwide trends and other NHS initiatives. In the second stage of the analysis we undertook a controlled regression analysis to estimate a within practice difference-in-difference effect of the intervention. Controls were selected at random from all practices classified as non-intervention practices (i.e. those practices not on the list provided by commercial companies). For computational reasons our analysis was restricted to data from all intervention practices and a random 10% sample of non-intervention practices, with between 778 and 976 control practices providing data at any one time.

For each continuous GPPS experience measure a separate mixed effects linear regression model was used. With the exception of changing the outcome (patient experience measure) the structure of the models is otherwise the same. Patient-level adjustment is made for self-reported gender, age (eight groups) and ethnicity (five groups) taken from GPPS responses and Index of Multiple Deprivation (IMD, a small area measure of socio-economic deprivation based on patient's postcode of residence) using groups defined by national quintiles. There is an indicator variable for survey wave capturing both seasonal differences and longer term trends. Variation in baseline levels for each practice resulting in clustering are accounted for using a random intercept for practice, as well as a random slope for time allowing for differential trends. This modelling allows us to capture the background scores against which

the effect of the intervention can be measured. Respondents who had not provided data for one or more of the socio-demographic variables were excluded from the analysis.

The effect of the intervention is captured using two fixed effect variables. The first is an indicator variable which takes the value 1 where the practice was an intervention practice and the survey was mailed after the intervention had started and zero otherwise. This term is intended to capture a step change in experience measure after starting the 'telephone first' approach. The second variable is equal to the time (in years) since the intervention started at a practice and survey mail out. Where the practice is a non-intervention practice, or the survey mail out precedes the intervention this variable equals zero. This variable is intended to capture a change in trend of patient experience scores post intervention. Further, random slopes are included for both of these variables to allow for heterogeneity in the effect of the intervention between practices. The estimated standard deviation of these random slopes is combined with the fixed effects to calculate a 95% mid-range, i.e. the range of intervention effect we expect to see across most practices.

Finally a supplementary analysis was performed to investigate if the effect of the intervention was differential between those in or not in work by including a main effect for working status (based on GPPS responses) and an interaction between working status and the intervention variable. This analysis was motivated by early findings from the qualitative workstream.

1.3. Methods of analysis of hospital utilisation data

A&E, Outpatient and Inpatient Hospital Episode Statistics (HES) data were obtained from NHS Digital covering the time period from April 2008 to March 2016.

A&E and Outpatient attendances are recorded at as attendances in HES, whereas inpatient HES data are recorded as a series of "Finished Consultant Episodes" (i.e. time spent under a particular consultant's care). Prior to analysis we link these episodes together to form single admissions using the University of York Centre for Health Economics Continuous Inpatient Spell (CIPS) definition. Admissions were flagged if they were related to an ambulatory sensitive condition (ACS) based on the classification used by Bardsley et al[3]. Further, admissions were defined as either elective or emergency admissions based on the HES data classification (a small number of admissions were defined as 'other' in the HES data and although they are included in the all admissions counts, they are not included in the elective or emergency counts).

[3] Bardsley M, Blunt I, Davies S, et al. Is secondary preventive care improving? Observational study of 10- year trends in emergency admissions for conditions amenable to ambulatory care. BMJ Open 2013; 3: e002007.

For all observations where a patient's general practice was recorded, patient age, gender, and index of multiple deprivation (IMD) (a measure of small area-level deprivation of the patient's home address) were extracted. Age groups were then defined as 5 year age groups up to the age of 19, then 10 year bands up to the age of 89 with a further group containing all ages 90 and over. IMD was classified into 5 groups with quintile defining cut points, from most to least deprived. The number of attendances/admissions per calendar month in each age group, by gender, and by IMD strata were calculated within each practice separately for each year and month of data for the following outcomes:

- A&E attendances
- Outpatient attendances
- All inpatient admissions
- All inpatient admissions for ambulatory care sensitive (ACS) conditions
- Elective inpatient admissions
- Emergency inpatient admissions

For computational reasons we did not use all practices in England as controls, but rather used a random 10% sample of practices in England which we did not believe had used a 'telephone first' approach. Based on denominator files of practice age and gender profiles extracted from the National Health Application and Infrastructure Services (NHAIS) systems annually from April 1st 2008 to April 1st 2015, we excluded all control practices from the initial sample where the list size was less than 1000 in at least one year of the study as very small practices are often atypical or may indicate new or closing practices. No intervention practices have a list size of <1000 at any time in the study period. We also excluded data from practices in years in which their practice code did not appear in the NHAIS system denominator files, even when attendances/admissions were attributed to patients at the practice. Further, we excluded the data from practices in the year preceding one where the practice did not appear, in order to exclude practices where mergers or closures may have occurred during the year of analysis. One intervention practice was excluded on the basis of this criteria, with a launch date in September, but the practice no longer appearing in the Exeter denominator files the following April. This gives a final analysis sample of 841 control practices and 145 intervention practices.

Finally, the data in intervention practices were restricted to between one-year before and one-year after the practice launch date. This was to focus any changes on those attributable to the 'telephone first' approach rather than any other factors, including potential concerns about the impact of practice mergers and closures. Data for control practices were included for the whole time period to give the best characterisation of background trends and variability.

In some months, practices included in the analysis had no relevant admissions or attendances for A&E, Inpatient or Outpatient data in one or more age, gender and IMD combination (there are 120 possible

combinations). For each of these practice months, where there were no attendances or admissions for a particular stratum, we include the strata with a count of “0” admissions or attendances.

A regression analysis was performed for each outcome looking first for within practice step changes at the time the intervention was introduced and second for a within practice change in the preceding trend (e.g. slowing down of a previous increase). We also model heterogeneity in these changes to examine whether the intervention has a different effect in different practices.

Our main analysis was conducted on an intention to treat basis. It includes all practices as identified by the commercial companies even when they informed us that the practices were running a hybrid form of the system or were no longer running the system. This was done in order to avoid selection bias, whereby only the successful practices continue with the system in the recommended form. A sensitivity analysis (appendix 3.5) was also performed restricting the analysis to practices where we believed, on the basis of information provided by the commercial companies, that the system was being run consistent with the companies’ protocols.

A mixed effects Poisson regression was used adjusting for patient age, gender and time period (96 dummy variables, one for each month from April 2008 to March 2016) as fixed effects, a random intercept to account for different baseline rates between practices, and a random slope for a continuous time variable allowing for different trends between practices. The effect of the intervention is captured using two fixed effect variables. The first is an indicator variable which takes the value 1 where the practice was an intervention practice and the HES data related to a time period after the intervention had started and zero otherwise. This term is intended to capture a step change in attendances after starting the ‘telephone first’ approach. The second variable is equal to the time (in years) since the intervention started at a practice. Where the practice is a non-intervention practice, or the data relates to a period preceding the intervention this variable equals zero. This variable is intended to capture a change in trend of patient experience scores post intervention. We additionally included a second time period variable with time pre- and post- intervention in intervention practices only, and 0 in control practices, to allow for a differential trend in admissions or attendances in control compared with intervention practices overall, unrelated to the intervention start date. Further, a random slope is included for the step change variable to allow for heterogeneity in the effect of the intervention between practices. The estimated standard deviation of these random slopes is combined with the fixed effects to calculate a 95% mid-range, i.e. the range of intervention effect we expect to see across most practices.

1.4. Methods of economic analysis

The methods of economic analysis are summarised in table C.

Table C. Summary of economic analyses

Item	Data sources	Approach to analysis
Primary Care Costs		
1. Cost of implementation and operation in intervention practices.	Telephone survey with intervention practices using structured telephone questionnaire	Costs reported as non-staff fixed and variable costs related to the implementation and routine operation of telephone first, and costs of any staffing changes.
2. Consultations	Data from 'GP Access' practices	Overall change in minutes spent consulting multiplied by cost per minute.
3. Prescribed medications by month by practice	GP Practice Prescribing data – all intervention practices and 829 randomly sampled control practices for 2008 to 2016	Analysis of quantity and cost of drugs monthly from 2008 to 2016 in control practices and +/- 1 year of commencement in intervention practices. Sub-analyses for ambulatory care-sensitive conditions & antibiotic prescriptions. 2016 price year. Multilevel mixed-effects log-linear regression adjusting for time period and intervention as fixed effects and random intercept and slope.
Secondary Care Costs	HES data - all intervention practices and 829 randomly sampled control practices for 2008 to 2016	Application of unit costs to calculate cost of A&E and outpatient attendances and inpatient admissions (all, elective and emergency, and for ambulatory care-sensitive conditions)

The telephone survey on the costs of the intervention was based on a previous survey undertaken by members of the research team[4]. The survey was administered in the twenty intervention practices taking part in the survey of patient experience. The questionnaire was completed in a single telephone interview with the practice manager or a colleague nominated by the practice manager, with

[4] Ling, T., et al. (2012). National Evaluation of the Department of Health's Integrated Care Pilots: Appendices, RAND Europe. http://www.rand.org/content/dam/rand/pubs/technical_reports/2012/RAND_TR1164.pdf

interviews conducted between March 2015 and December 2016. The questionnaire was divided into two parts, staff and non-staff costs. Costs are reported as stated by respondents, inclusive of VAT (at 20%) where applicable and adjusted where necessary to 2015/16 price year using the Hospital and Community Health Services inflation index, with staffing costs estimated based on the mid-point of the respective staff grade for 2015/16[5]. Respondents were invited to indicate the level of certainty about cost estimates provided on a scale of 1 (least certain) to 10 (most certain). Changes in total consultation time per day by practice GPs reported were costed at national 2015/16 prices[5], assuming an 8-hour working day.

Data on prescribed medications were extracted from GP Practice Prescribing data month by month from 2008-2016 in the 829 randomly sampled control practices used in the HES analysis (described above) and for +/- 12 months of launch date of telephone first in intervention practices. GP practice prescribing data[6] lists the quantities and costs of drugs dispensed in the community in England by GP practice. Data on all items, and items commonly prescribed for ambulatory care-sensitive conditions[7] and for antibiotics were summarised by month. Net Ingredient Costs (NIC) reported in the data were inflated to 2016 prices using the pharmaceutical products portion (section 06.1.1) of the consumer prices index (CPI) for drugs reported under BNF chapters 1 to 19, and the other medical and therapeutic equipment portion (section 06.1.2/3) of the CPI for items within BNF chapters 20-23[8,9]. NICs are based on the list price of drugs and so exclude any discount or local price negotiation offered to community pharmacies (details of these are not generally made public). The NIC also excludes dispensing fees and does not adjust for whether or not a prescription charge was paid by the patient. Analyses were conducted for all prescriptions, for selected ambulatory care sensitive conditions and antibiotics.

Hospital Episode Statistics (HES) data were obtained from NHS Digital between April 2008 and March 2016 for intervention and control practices as described for hospital activity data (above). The field 'SUSHRG' (secondary uses service health resource group) was matched to the relevant HRG 2015/16 reference cost for A&E, outpatient contacts and inpatient episodes of care (finished consultant

---

[5] Curtis, L. and A. Burns (2015). Unit Costs of Health and Social Care 2015. Canterbury, University of Kent. *www.pssru.ac.uk/project-pages/unit-costs/2015/*

[6] https://data.gov.uk/dataset/prescribing-by-gp-practice-presentation-level.

[7] Based on lists in: Tan Y, Dixon A, Gao H. Data Briefing April 2012. Emergency hospital admissions for ambulatory care-sensitive conditions: identifying the potential reductions. https://www.kingsfund.org.uk/sites/files/kf/field/field_publication_file/data-briefing-emergency-hospital-admissions-for-ambulatory-care-sensitive-conditions-apr-2012.pdf.

[8] https://www.ons.gov.uk/economy/inflationandpriceindices#tab-data-tables.

[9] Office for National Statistics. Inflation and Price indices. https://www.ons.gov.uk/economy/inflationandpriceindices#tab-data-tables.

episodes) [10]. Where there was no valid HRG, a weighted average cost of the HRG stem (e.g. AA23) or HRG root (e.g. AA) was applied. Where there was still no valid HRG, an average unit cost for an A&E, outpatient or inpatient cost[11] was applied. The cost of referrals per 1000 patients in intervention practices was then calculated for 12 months prior to and post introduction of telephone first, and the crude change calculated. The step change attributable to telephone first was calculated by multiplying the costs in the 12 months prior by RR – 1, where RR is the proportionate step change reported in Table 3 in the main paper. The slope change attributable was then calculated by multiplying the cost in the 12 months prior by exp(ln(RR)/2) – 1, where RR is the proportionate slope change reported in Table 3. The total change is the sum of the two. These changes allow for background trends in the random 10% sample of control practices in England.

[10] Department of Health. NHS Reference costs 2015/16. https://www.gov.uk/government/publications/nhs-reference-costs-2015-to-2016

[11] Department of Health. Reference costs 2015/16. https://www.gov.uk/government/uploads/system/uploads/attachment_data/file/577083/Reference_Costs_2015-16.pdf
