# Supplementary material for: Evaluation of telephone first approach to demand management in English general practice: observational study
Source: BMJ. 2017 Sep 28;358:j4197. doi: 10.1136/bmj.j4197 (PMC5615264; doi:10.1136/bmj.j4197)
Supplement: Supplementary file 2 — Appendix 2: Patient questionnaire [file newj040067.ww2.pdf]

Appendix 2. Patient questionnaire [posted as supplied by author]

Tele-First: Telephone Triage in General Practice

QUESTIONNAIRE: Speaking to a GP on the phone before arranging an appointment

As we've explained in the attached letter, you've been sent this questionnaire because you recently spoke to a GP on the phone when you asked for an appointment with a doctor or nurse at your GP surgery.

Please complete this questionnaire and return it to us in the Freepost envelope provided.

If you're completing the questionnaire because you spoke to the GP on behalf of someone else, please answer all the questions based on the care they received.

Please contact us if you have any questions or queries on: telephonetriage@rand.org, or by phone at 01223 353xxx.

Some questions about the telephone appointment

Please complete the following questions about your contact with the GP surgery on the date referred to in the attached letter.

1. Who was the patient whose health was discussed in the telephone call? (*Please tick one box only*)

☐ Me ☐ My child ☐ An adult for whom I am carer ☐ Other *(Please specify)* ____________________

2. Who spoke to the doctor on the telephone on that day? (*Please tick all that apply*)

☐ The patient ☐ The patient's parent/guardian ☐ The patient's carer ☐ Other *(Please specify)* ____________________

3. How did you first contact the practice that day to ask for an appointment? (*Please tick one box only*)

☐ Telephone ☐ Online ☐ In person ☐ Other *(Please specify)* ____________________

4. Before you contacted the practice, were you expecting to receive a telephone call back from your GP? (*Please tick one box only*)

☐ Yes ☐ No ☐ Not sure
☐

5a. How long did it take for a GP to call you back? (*Please tick one box only*)

☐ Less than 20 minutes ☐ 20 - 60 minutes ☐ More than 1 hour ☐ GP did not call back

5b. How do you rate this? (*Please tick one box only*)

☐ Very poor ☐ Poor ☐ Acceptable ☐ Good ☐ Excellent

6a. What was the outcome of the telephone call? (*Please tick all that apply*)

☐ I received telephone advice only, no further follow-up

☐ I was given a prescription

☐ An appointment with a GP in the surgery was arranged

☐ An appointment with a nurse in the surgery was arranged

☐ A follow-up telephone appointment with a GP was arranged

☐ A follow-up telephone appointment with a nurse was arranged

☐ Other (*Please specify):* ____________________

6b. Were you satisfied with this outcome? (*Please tick one box only*)

☐ Yes, I was satisfied

☐ No, I thought I should have had a face-to-face appointment in the surgery

☐ No, I thought a follow-up telephone appointment should have been arranged

☐ Other *(Please specify):* ____________________

Your opinions on the telephone appointment

7. **Did you find the telephone appointment more or less convenient than just attending a face-to-face appointment, without being able to talk to the doctor on the phone first?** (*Please tick one box only*)

☐ More convenient ☐ Less convenient ☐ No difference

8. **Did you find it more or less difficult to communicate with the GP over the phone than in person?** (*Please tick one box only*)

☐ More difficult ☐ Less difficult *(Please go to question 10)* ☐ No difference *(Please go to question 10)*

9. If you answered 'More difficult' to the previous question, why do you think it was more difficult to communicate over the phone? (*Please tick all that apply*)

- ☐ English is not my first language
- ☐ The doctor really needed to see me
- ☐ The telephone line was not clear
- ☐ I have impaired hearing
- ☐ I found it difficult to explain the problem
- ☐ Other *(Please specify)* ____

If you wish to give more detail, please add additional comments at the end of the form.

Some questions about the patient

We would now like to ask some questions to help us see how experiences of telephone appointments vary between different groups of people.

If you are completing the questionnaire for someone else, please answer the questions about the person who was the patient.

10. Are you (or the patient, if you are responding on his or her behalf) male or female?

- ☐ Male
- ☐ Female

11. How old are you (or is the patient, if you are responding on his or her behalf)?

- ☐ Under 18
- ☐ 18 to 24
- ☐ 25 to 34
- ☐ 35 to 44
- ☐ 45 to 54
- ☐ 55 to 64
- ☐ 65 to 74
- ☐ 75 to 84
- ☐ 85 or over

12. In general, would you say your health (or that of the patient, if you are responding on his or her behalf) is…?

- ☐ Excellent
- ☐ Very good
- ☐ Good
- ☐ Fair
- ☐ Poor

13. Do you (or the patient if you are responding on his or her behalf) have any long-standing illness, disability or infirmity? By long-standing I mean anything that has troubled you over a period of time, or that is likely to affect you over a period of time.

- ☐ Yes
- ☐ No

14. What is your ethnic group (or that of the patient if you are responding on his or her behalf)? *(Please choose one option that best describes your ethnic group or background)*

A. White

- ☐ English / Welsh / Scottish / Northern Irish / British
- ☐ Irish
- ☐ Gypsy or Irish Traveller
- ☐ Any other White background, *please describe:* ____

B. Mixed / Multiple ethnic groups

- ☐ White and Black Caribbean
- ☐ White and Black African
- ☐ White and Asian
- ☐ Any other Mixed/Multiple ethnic background, *please describe:* ____________________________________

C. Asian / Asian British

- ☐ Indian
- ☐ Pakistani
- ☐ Bangladeshi
- ☐ Chinese
- ☐ Any other Asian background, *please describe:* ____

D. Black / African / Caribbean / Black British

- ☐ African
- ☐ Caribbean
- ☐ Any other Black / African / Caribbean background, *please describe:* _______________________________

E. Other ethnic group

- ☐ Arab
- ☐ Any other ethnic group, *please describe*: ____

15. What is the main language you (or the patient, if you are responding on his or her behalf) speak/s at home?

- ☐ English *(go to question 17)*
- ☐ Other, *write in (including British Sign Language)*

____

16. If you answered 'Other' to the previous question, can you tell us how well you (or the patient, if you are responding on his/her behalf) can speak English?

☐ Very well ☐ Well ☐ Not well ☐ Not at all

17. If you (or the patient, if you are responding on his or her behalf) need to see a GP at the GP surgery during typical working hours, can you take time away from your work to do this?

☐ Yes, I can easily take time away from work

☐ Yes, I can take time away from work but with some difficulty

☐ No, I can't take time away from work

☐ Not relevant as I do not work (child, unemployed, retired)

Overall satisfaction with the telephone appointment system

18. **Would you like to go back to the old system, where most GP appointments were face to face?**

☐ Yes ☐ No ☐ Don't mind

Please add any additional comments in the box on the next page.

Thank you very much for taking the time to fill out this questionnaire.

Please return in enclosed Freepost envelope – no stamp required.

Tele-First Project Team, RAND Europe, Westbrook Centre, Milton Road, Cambridge CB4 1YG

Please add any additional comments here

We would like to contact a small number of people to interview in more detail about their experiences with telephone consultation. If you would be happy to be contacted, please provide your contact details and tick the box below.

☐ I am happy to be contacted for an interview

Name:____

Telephone:____

Email address:____

Please underline the method that you would prefer us to use for contacting you.

119_1100_1
