# Supplementary material for: Evaluation of telephone first approach to demand management in English general practice: observational study
Source: BMJ. 2017 Sep 28;358:j4197. doi: 10.1136/bmj.j4197 (PMC5615264; doi:10.1136/bmj.j4197)
Supplement: Supplementary file 3 — Appendix 3: Further details of results [file newj040067.ww3.pdf]

Appendix 3. Further details of results [posted as supplied by author]

3.1 Results on primary care utilisation: super-posed epoch analyses

The following data show the intention to treat analysis in greater detail, including super-posed epoch graphs which show the change in outcomes for each individual practice (black lines) and the mean value (red lines). For each individual practice the date the 'telephone first' approach was launched was set at time zero. These graphs illustrate both the mean values, but also the wide variation between individual practices. For each outcome, we also show the results of the regression analysis (see appendix 1 for details). Figures A and B are also included in the main paper for illustrative purposes.

<u>Numbers of appointments: total, face-to-face- and telephone</u>

The mean number of appointments per 1,000 patients per day was 16.5 (SD 6.3) before the intervention started increasing to 21.8 (SD 8.1) post intervention (fig A). This increase differed by appointment type with decreases seen in the number of face to face appointments from mean=13.0, SD= 4.5, to mean=9.3, SD=5.5 (fig B) and increases in the number of telephone appointments from mean=3.0, SD=4.0, to mean=12.2, SD=7.5 (fig C).

Figure A Super posed epoch analysis showing the change in total number of appointments per 1,000 patients per day relative to the intervention launch

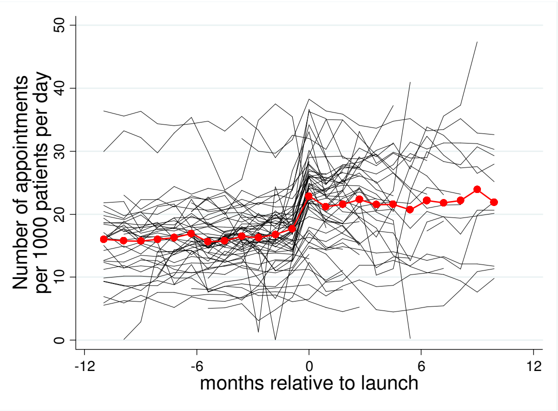

NOTE: The black lines represent the mean within a single practice relative to launch time, with each black line representing a single intervention practice. The red dots represent the mean of individual practice means.

Figure B Super posed epoch analysis showing the change in the number of face-to-face appointments per 1,000 patients per day relative to the intervention launch

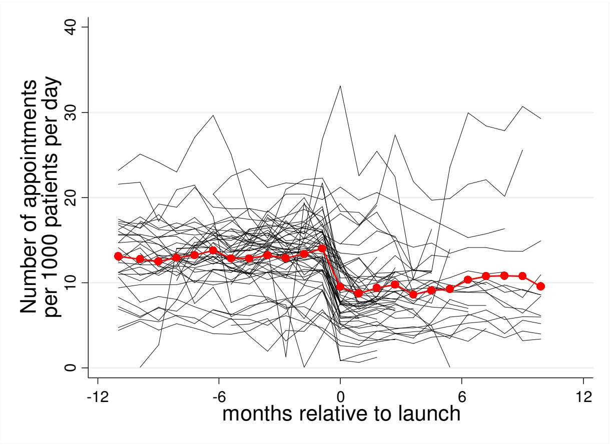

Figure C Super posed epoch analysis showing the change in the number of telephone appointments per 1,000 patients per day relative to the intervention launch

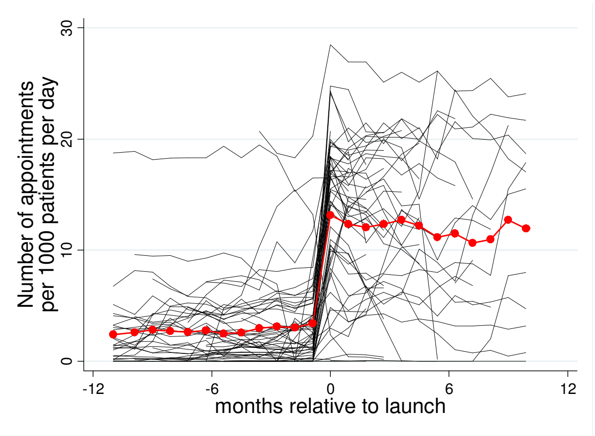

<u>Time waited for an appointment</u>

The average number of days between booking an appointment and having an appointment across all intervention practices was 4.0 (SD 7.0) prior to the intervention and 0.9 (SD 3.9) after the intervention. Similar drops in time between booking and having an appointment are seen when restricting to face to face appointments (from mean=4.5 days, SD=7.4, to mean=1.8 days, SD=5.6 days) and telephone appointments (from mean=1.8 days, SD=4.2, to mean=0.3 days, SD=1.7 days).

Figure D Super posed epoch analysis showing the change in mean time between booking and having an appointment of any type relative to the intervention launch

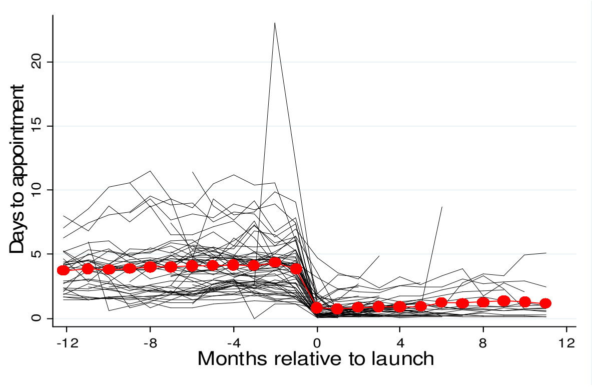

<u>Length of appointment</u>

On average the length of appointment decreased from 10.5 minutes (SD 6.0) minutes pre intervention to 8.5 minutes (SD 6.2) minutes post intervention (figure E). The change in length was smaller when restricting to face to face appointments (from mean=10.9 minutes, SD=5.9, to mean=10.2 minutes, SD=6.4) or telephone appointments (from mean=7.7 minutes, SD= 6.0, to mean=6.2 minutes, SD=5.1) suggesting that much of the overall reduction in average appointment length is due to a change in the proportion of appointments that are telephone appointments. It is worth bearing in mind that patients who had a face to face appointment may also have had a telephone appointment. As such it is likely that the total consultation length for each patient increases for patients who had both phone and face to face appointments. However, as we cannot link telephone and face to face appointments, we cannot demonstrate this is actually the case.

Figure E Super posed epoch analysis showing the change in appointment length relative to the intervention launch

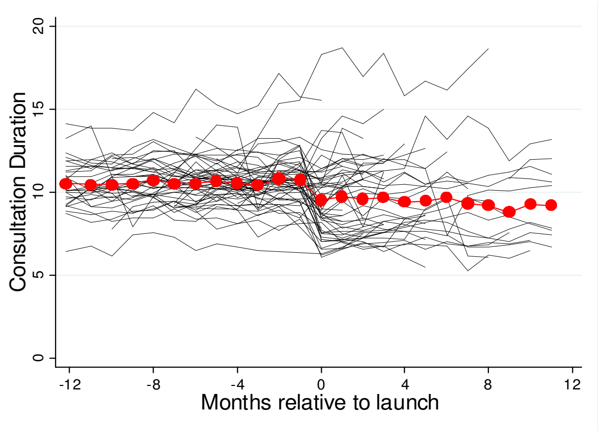

Total time spent consulting per day by practice GPs

The mean time spent consulting per 1000 patients increased slightly from 7.8 (SD 2.5) hours per day pre intervention to 8.5 (SD 4.0) post intervention (fig F). It should be noted that the number of practices providing data decreases rapidly in the months after launch and so medium term effects may be hidden by this.

Figure F Super posed epoch analysis showing the change in total time spent consulting relative to the intervention launch – hours per 1000 patients per day

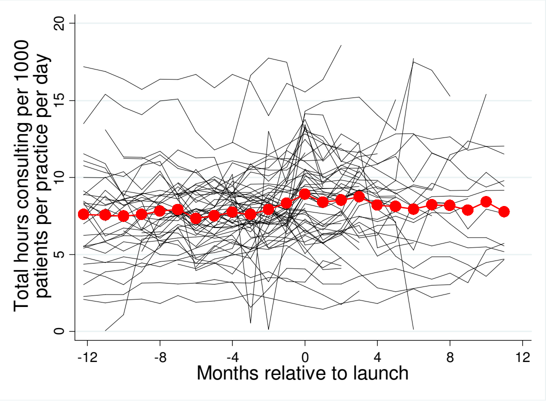

Continuity of care

The mean continuity index (which takes possible values between 0 and 1) in practices was very similar before and after the launch of the intervention at 0.728 (SD 0.077) and 0.731 (SD 0.79) respectively. This can be seen in the superposed epoch analysis (fig G) where there is some fluctuation over time but roughly equal before and after launch. It is also worth noting that the data becomes sparse more than five months post launch.

Figure G Super posed epoch analysis showing the continuity index relative to the months to launch

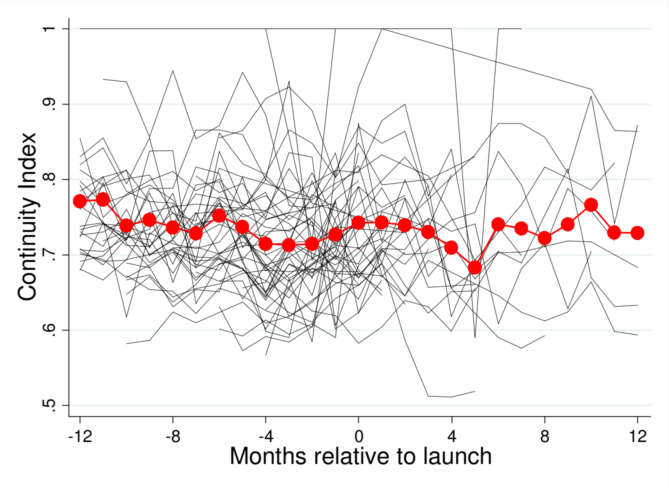

NOTE: The black lines represent the mean within a single practice relative to launch time, with each black line representing a single intervention practice. The red dots represent the mean of individual practice means.

3.2. Primary care utilisation: regression analysis

Table A shows there was a 28% increase in the numbers of all appointments following the introduction of the intervention (rate ratio [RR] at step change 1.28, 95% CI 1.17 to 1.39, p<0.0001), which comprised a 38% drop in face to face appointments and a 12-fold increase in the number of telephone (see rate ratios for step change in table, p<0.0001 for all). It should be noted that the large relative change in telephone appointments in part reflects small numbers pre-launch. Although there was a slight slowing in the initial rate of increase, the total number of appointments continued to increase post intervention by 4% per year (RR 1.04, 95% CI 1.04 to 1.05, p<0.0001). There was considerable heterogeneity between practices in the changes in total number of appointments with the 95% mid-ranges suggesting that some practices reduced overall appointment numbers by up to 32% whilst for others the total number of appointments increased by a factor of 2.4 times. The per protocol sensitivity analysis produced broadly consistent findings (appendix 3.3).

Table A Results of mixed effects Poisson regression showing the effect of the intervention on the number of appointments. Adjustment is also made for month and day of week as well as a random intercept for practice to account for different baseline level

Appointment type	Step change at transition			Pre-transition trend		Post- transition trend		Interaction p-value**
	Rate ratio	p-value	Heterogeneity*	Rate ratio	p-value	Rate ratio	p-value	
All	1.28 (1.17, 1.39)	<0.0001	0.68, 2.39	1.07 (1.06, 1.08)	<0.0001	1.04 (1.04, 1.05)	<0.0001	<0.0001
Face to face	0.62 (0.55, 0.71)	<0.0001	0.24, 1.62	1.03 (1.01, 1.04)	<0.0001	0.98 (0.97, 0.99)	<0.0001	<0.0001
Telephone	12.04 (6.33, 22.90)	<0.0001	0.10, 1467.39	1.11 (1.09, 1.12)	<0.0001	1.46 (1.43, 1.49)	<0.0001	<0.0001

* The heterogeneity is given in terms of the 95% mid-range for practices. This is the range of rate ratios for the "true" step changes we expect to see across 95% of practices after accounting for patient sample size. This has been estimated using the standard deviation of the random slope for the step change (σ_{step}) combined with the fixed effect of the step change (β_{step}) as $e^{\beta_{step} \pm 1.96\sigma_{step}}$.
**Interaction p-value is for a test of whether the post-transition trend is different to the pre-transition trend.

Table B shows the time between booking and having an appointment dropped, on average, by 3.3 days (95% CI -3.8 to -2.8, p<0.0001) after initially switching to the intervention (step-change). The same decrease is seen for face to face appointments and a smaller reduction is seen for telephone consultations. The time between booking and having an appointment continued to increase over time following the introduction of the intervention. However, given the annual increase in time waited is much smaller than the initial step decrease in time waited following the introduction of the intervention, it would take many years for the waiting time in the average practice to return to pre-intervention levels. Again the 95% mid-ranges show that was substantial heterogeneity between practices in the reduction in length of time waited between booking and having an appointment, however, all practices show a decrease in the time between booking and having an appointment.

Table B Results of mixed effects linear regression showing the effect of the intervention on the time between booking and having an appointment in days. Adjustment is also made for month and day of week as well as a random intercept for practice to account for different baseline level

Appointment type	Step change at transition			Pre-transition trend		Post- transition trend		Interaction p-value**
	Time to appointment	p-value	Heterogeneity*	Change in time to appointment in days/year	p-value	Change in time to appointment in days/year	p-value	
All	-3.30 (-3.80, -2.80)	<0.0001	-5.91, -0.71	0.20 (0.16, 0.25)	<0.0001	0.22 (0.16, 0.27)	<0.0001	0.6928
Face to face	-3.31 (-3.89, -2.72)	<0.0001	-6.31, -0.31	0.53 (0.47, 0.59)	<0.0001	0.49 (0.40, 0.58)	<0.0001	0.4982
Telephone	-0.78 (-1.07, -0.48)	<0.0001	-2.21, 0.71	-0.21 (-0.26, -0.16)	<0.0001	0.15 (0.12, 0.18)	<0.0001	<.0001

* The heterogeneity is given in terms of the 95% mid-range for practices. This is the range of differences for the "true" step changes we expect to see across 95% of practices after accounting for patient sample size. This has been estimated using the standard deviation of the random slope for the step change (σ_{step}) combined with the fixed effect of the step change (β_{step}) as $\beta_{step} \pm 1.96\sigma_{step}$
**Interaction p-value is for a test of whether the post-transition trend is different to the pre-transition trend.

Table C shows the regression analysis for length of consultation and suggests that the differences observed in the superposed epoch analysis (appendix 3.1) exaggerate the effect of the intervention somewhat. Across all appointment types there was an average decrease in appointment length of 0.9 minutes (95% CI -1.43 to -0.33, p=0.0024) immediately following the introduction of the intervention. There was no significant evidence of a change in the duration of face to face appointments (p=0.18), whilst telephone appointments decreased in duration by about half a minute. There 95% mid-ranges indicate that there was considerably heterogeneity between practices, with some practices reducing the length of appointments and others increasing the appointment lengths. For all appointments there was a reversal in the pre-intervention trend of increasing appointment length to a post-intervention trend of decreasing appointment length such that, on average, over the year following intervention launch there was an additional reduction of around 0.3 minutes.

Table C Results of mixed effects linear regression showing the effect of the intervention on the duration of appointments. Adjustment is also made for month and day of week as well as a random intercept for practice to account for different baseline level

Appointment type	Step change at transition			Pre-transition trend		Post- transition trend		Interaction p-value*
	Change in length of appointment (minutes)	p-value	Heterogeneity*	Change in length of appointment (minutes/year)	p-value	Change in length of appointment (minutes/year)	p-value	
All	-0.88 (-1.43, -0.33)	0.0024	-3.67, 1.91	0.19 (0.14, 0.25)	<0.0001	-0.28 (-0.35, -0.21)	<0.0001	<0.0001
Face to face	0.22 (-0.11, 0.55)	0.1800	-1.41, 1.86	0.35 (0.29, 0.41)	<0.0001	-0.34 (-0.43, -0.24)	<0.0001	<0.0001
Telephone	-0.51 (-0.89, -0.13)	0.0096	-1.79, 0.77	0.42 (0.28, 0.57)	<0.0001	-0.39 (-0.50, -0.29)	<0.0001	<0.0001

* The heterogeneity is given in terms of the 95% mid-range for practices. This is the range of differences for the "true" step changes we expect to see across 95% of practices after accounting for patient sample size. This has been estimated using the standard deviation of the random slope for the step change (σ_{step}) combined with the fixed effect of the step change (β_{step}) as $\beta_{step} \pm 1.96\sigma_{step}$
**Interaction p-value is for a test of whether the post-transition trend is different to the pre-transition trend.

Table D shows the regression analysis for continuity of care and provides evidence (p<0.001) that continuity improves after starting the intervention but only by a small amount (difference of 0.05, 95% CI 0.04, 0.08 on a scale between 0 and 1). Although there is evidence of a subsequent decrease in continuity it would take around 10 years to erode the initial gains made if this decline were to continue. As with other outcomes there is strong heterogeneity in the effect of the intervention between practices with continuity increasing in most practices, but decreasing in some (illustrated by the 95% mid-range shown in table D.

Table D Results of mixed effects linear regression showing the effect of the intervention on the usual provider continuity index for face to face appointments. Adjustment is also made for month, patient age as well as a random intercept for practice to account for different baseline levels.

Step change at transition			Pre-transition trend		Post- transition trend		Interaction
**Change in continuity index**	**p-value**	**Heterogeneity***	**Change in continuity index per year**	**p-value**	**Change in continuity index per year**	**p-value**	**p-value***
0.058 (0.037, 0.081)	<0.001	-0.074, 0.191	-0.0001 (-0.0002, 0.0001)	0.40	-0.006 (-0.006, -0.005)	<0.001	<0.001

* The heterogeneity is given in terms of the 95% mid-range for practices. This is the range of differences for the "true" step changes we expect to see across 95% of practices after accounting for patient sample size. This has been estimated using the standard deviation of the random slope for the step change (σ_{step}) combined with the fixed effect of the step change (β_{step}) as $\beta_{step} \pm 1.96\sigma_{step}$
**Interaction p-value is for a test of whether the post-transition trend is different to the pre-transition trend.

For total time spent consulting, table E shows here was only very weak evidence (p=0.088) of any change to the total time spent consulting by GPs each day with, on average, an estimated increase of 8% (ratio of time 1.08 95% CI 0.99, 1.17). Consistent with the analysis on number of appointments the total time spent consulting in face to face appointments fell and increased for telephone appointments. As with other outcomes large heterogeneity in the effect was seen such as some practices experienced an increase in the total time spent consulting of up to 79% while others saw decreases up to 35%, as illustrated by the 95% mid-range.

Table E Results of mixed effects linear regression showing the effect of the intervention on the total time spent consulting*.

Appointment type	Step change at transition			Pre-transition trend		Post- transition trend		Interaction
	**Ratio**	**p-value**	**Heterogeneity****	**Ratio**	**p-value**	**Ratio**	**p-value**	**p-value*****
All	1.08 (0.99, 1.17)	0.0883	0.65, 1.79	1.00 (0.94, 1.05)	0.8728	1.05 (1.02, 1.09)	0.0051	0.0856
Face to face only	0.60 (0.52, 0.70)	<0.0001	0.23, 1.57	1.09 (1.03, 1.14)	0.0018	0.99 (0.96, 1.03)	0.6398	0.0045
Telephone	5.45 (3.41, 8.72)	<0.0001	0.54, 55.6	0.97 (0.87, 1.08)	0.5921	1.50 (1.38, 1.63)	<0.0001	<0.0001

*Adjustment is also made for month and day of week as well as a random intercept for practice to account for different baseline level. Exponetiated regression coefficients are shown which represent a relative change (ratio) in time spent consulting. Standard errors are corrected in the adjusted for missing data analyses
** The heterogeneity is given in terms of the 95% mid-range for practices. This is the range of ratios for the "true" step changes we expect to see across 95% of practices after accounting for patient sample size. This has been estimated using the standard deviation of the random slope for the step change (σ_{step}) combined with the fixed effect of the step change (β_{step}) as $e^{\beta_{step} \pm 1.96\sigma_{step}}$.
***Interaction p-value is for a test of whether the post-transition trend is different to the pre-transition trend.

3.3 Primary care utilisation: per-protocol sensitivity analysis

This appendix presents findings from the sensitivity analysis for the analysis of appointment data. The sensitivity analysis followed the same methods as those reported in appendix 3.2 but the data were restricted to practices where we believed, on the basis of information provided by the commercial company, that the system was being run consistent with the company's protocols. The sensitivity analysis included data from 27 practices covering 997,772 appointments over 8,158 practice days.

Number of appointments

Table F Results of mixed effects Poisson regression showing the effect of the intervention on the number of appointments. Adjustment is also made for month and day of week as well as a random intercept for practice to account for different baseline level

Appointment type	Step change at transition		Pre-transition		Post- transition		Interaction p-value*	95% mid-range for step change transition
	IRR	p-value	IRR	p-value	IRR	p-value		
All appointments	1.28 (1.13, 1.46)	<0.001	1.10 (1.09, 1.11)	<0.0001	1.02 (1.01, 1.04)	<0.0001	<0.0001	0.66, 2.47
Face to face	0.54 (0.42, 0.68)	<0.0001	1.03 (1.01, 1.05)	0.003	0.97 (0.96, 0.99)	<0.0001	<0.0001	0.16, 1.82
Telephone	14.35 (5.52, 37.30)	<0.0001	1.14 (1.13, 1.16)	<0.0001	1.30 (1.27, 1.34)	<0.0001	<0.0001	0.12, 1740.59

NOTE: *Interaction p-value is for a test of whether the post-transition trend is different to the pre-transition trend.

Time waited for an appointment

Table G shows that the per protocol sensitivity analysis produced broadly consistent findings for the step change in time waited immediately following the introduction of the intervention, but compared to the super-posed epoch analysis, there was evidence that the time waited between booking and getting an appointment of any type continued to decrease slightly post-intervention in these practices.

Table G Results of mixed effects linear regression showing the effect of the intervention on the time between booking and having an appointment. Adjustment is also made for month and day of week as well as a random intercept for practice to account for different baseline level

Appointment type	Step change at transition		Pre-transition trend		Post- transition trend		Interaction p-value*	95% mid-range for step change transition
	Time to appointment	p-value	Change in time to appointment in days/year	p-value	Change in time to appointment in days/year	p-value		
All	-3.48 (-4.11, -2.84)	<0.0001	-0.14 (0.07, 0.20)	<0.0001	-0.13 (-0.18, -0.07)	<0.0001	<0.0001	-5.71, -1.31
Face to face	-3.67 (-4.55, -2.79)	<0.0001	0.73 (0.63, 0.82)	<0.0001	-0.10 (-0.22, 0.01)	0.0665	<0.0001	-6.71, -0.61
Telephone	-0.62 (-1.10, -0.15)	0.0123	-0.34 (-0.40, -0.28)	<0.0001	-0.00 (-0.04, 0.03)	0.9246	<0.0001	-2.11, 0.91

NOTE: *Interaction p-value is for a test of whether the post-transition trend is different to the pre-transition trend.

Length of appointment, total time spent consulting, continuity of care

Table H shows the per protocol sensitivity analysis produced estimates in the same direction as the intention to treat analysis, but the decrease in average appointment length was smaller. This combined with larger standard errors due to the decreased sample size meant that the decrease in appointment duration was no longer statistically significant, either for all appointments or for telephone consultations.

Further results for total time spent consulting and continuity of care are shown in tables I and J. These results are broadly similar to the intention to treat analysis, though with weaker evidence of an increase in total time consulting (ratio 1.07 vs 1.08 with wider confidence intervals due to the decreased sample size).

Table H Results of mixed effects linear regression showing the effect of the intervention on the length of appointments. Adjustment is also made for month and day of week as well as a random intercept for practice to account for different baseline level

Appointment type	Step change at transition		Pre-transition trend		Post- transition trend		Interaction p-value*	95% mid-range for step change transition
	Change in length of appointment (minutes)	p-value	Change in length of appointment (minutes/year)	p-value	Change in length of appointment (minutes/year)	p-value		
All	-0.39 (-1.42, 0.64)	0.4414	0.30 (0.20, 0.39)	<0.0001	-0.85 (-0.95, -0.75)	<0.0001	<0.0001	-3.68, 2.90
Face to face only	0.84 (0.28, 1.41)	0.0052	0.47 (0.37, 0.58)	<0.0001	-1.03 (-1.16, -0.90)	<0.0001	<0.0001	-0.91, 2.59
Telephone	-0.66 (-1.53, 0.22)	0.1244	0.53 (0.34, 0.73)	<0.0001	-0.67 (-0.80, -0.54)	<0.0001	<0.0001	-2.08, 0.76

NOTE: *Interaction p-value is for a test of whether the post-transition trend is different to the pre-transition trend.

Table I Results of mixed effects linear regression showing the effect of the intervention on the total time spent consulting. Adjustment is also made for month and day of week as well as a random intercept for practice to account for different baseline level. SE corrected in the adjusted for missing data analyses

Appointment type	Step change at transition		Pre-transition trend		Post- transition trend		Interaction p-value*	95% mid-range for step change transition
	Ratio	p-value	Ratio	p-value	Ratio	p-value		
All	1.07 (0.93, 1.24)	0.3369	1.04 (0.97, 1.12)	0.2587	1.08 (1.01, 1.15)	0.0285	0.4841	0.62, 1.86
Face to face only	0.53 (0.40, 0.70)	<0.0001	1.11 (1.04, 1.19)	0.0021	1.00 (0.94, 1.07)	0.9625	0.0238	0.15, 1.82
Telephone	6.15 (3.08, 12.29)	<0.0001	1.12 (.98, 1.28)	0.0863	1.35 (1.18, 1.54)	<0.0001	0.0478	0.74, 51.37

NOTE: *Interaction p-value is for a test of whether the post-transition trend is different to the pre-transition trend.

Table J Results of mixed effects linear regression showing the effect of the intervention on the usual provider continuity index for face to face appointments. Adjustment is also made for month, patient age as well as a random intercept for practice to account for different baseline level.

Step change at transition		Pre-transition trend		Post- transition trend*		Interaction p-value	95% mid-range for step change transition
Change in continuity index	p-value	Change in continuity index per year	p-value	Change in continuity index per year	p-value		
0.067 (0.029, 0.105)	<0.001	0.0003 (0.0000, 0.0007)	0.072	-0.006 (-0.007, -0.006)	<0.001	<0.001	-0.103, 0.237

NOTE: *Interaction p-value is for a test of whether the post-transition trend is different to the pre-transition trend

3.4 Comparison with GP-Patient survey: per-protocol sensitivity analysis

This appendix presents findings from the sensitivity analysis for the GP Patient Survey data. The sensitivity analysis followed the same methods as those reported in the main paper but the data was restricted to practices where, to the best of our knowledge, the system was being run consistent with the method proposed by the commercial companies i.e. practices that are no longer using a 'telephone first' approach or practices running a 'hybrid' system were removed from the analysis. The results are very similar to those presented in the main paper with a large improvement in patients' rating of time to be seen and small decreases in patients recommending their surgery to others and to their ability to see a GP of their choice. However, as with the main analyses, initial decreases in reported experience for these last two showed some evidence of recovery over subsequent years.

***Table K** 'Per protocol' analysis of GP Patient Survey scores.*

GPPS outcome	Step change following intervention (95% CI)	p	Additional yearly change following intervention (95% CI)	p
Continuous outcomes – difference on 0-100 scale				
GP Communication Composite	**-1.36 (-2.01, -0.71)**	**<0.001**	0.16 (-0.16, 0.47)	0.33
Ease of getting through on the phone	1.79 (0.44, 3.13)	0.009	0.41 (-0.51, 1.33)	0.39
Would you recommend your GP surgery	**-2.70 (-3.77, -1.63)**	**<0.001**	0.24 (-0.34, 0.82)	0.42
Seeing preferred GP	**-1.75 (-3.32, -0.18)**	**0.03**	0.37 (-0.48, 1.22)	0.39
Time until seen or spoken to	**21.27 (18.87, 23.67)**	**<0.001**	1.91 (0.90, 2.93)	<0.001
Convenience of appointment	0.69 (-0.19, 1.57)	0.12	0.70 (0.33, 1.07)	<0.001
Overall experience of making an appointment	0.29 (-1.03, 1.61)	0.67	0.98 (0.33, 1.63)	0.003

3.5 Per-protocol sensitivity analysis of secondary care data

This section presents findings from the sensitivity analysis for the secondary care data, restricting the analysis to practices which we believed, on the basis of information provided by the commercial companies, were running the 'telephone first' approach consistent with the companies' protocols. The sensitivity analysis followed the same methods as those reported in the main paper. The results are very similar to those presented in the main report with no evidence of a step change in A&E and outpatient attendances, but evidence of a slower increase over time in intervention practices than the background trend. We also find evidence of step increases in inpatient admissions.

Table L Results of controlled mixed effect Poisson regressions modelling, adjusting for patient demographics, national seasonal and long term trend effects, clustering by practice including heterogeneity in baseline scores and trends.

Outcome	Step change at transition			Additional yearly change following intervention	
	Rate ratio (95% CI)	p-value	Heterogeneity*	Rate ratio per year (95% CI)	p-value
A&E attendances	1.00 (0.99 - 1.02)	0.71	0.92 - 1.10	0.98 (0.96 - 1.00)	0.012
Outpatient attendances	0.99 (0.98 - 1.00)	0.19	0.91 - 1.08	0.97 (0.96 - 0.97)	<0.0001
All inpatient admissions	1.00 (1.01 - 1.04)	0.0013	0.97 - 1.08	1.02 (1.00 - 1.03)	0.1
ACS inpatient admissions	1.05 (1.01 - 1.10)	0.026	0.87 - 1.27	1.10 (1.04 - 1.17)	0.0009
Elective inpatient admissions	1.03 (1.01 - 1.05)	0.0082	0.93 – 1.14	1.04 (1.02 - 1.07)	0.0012
Emergency inpatient admissions	1.02 (1.00 - 1.04)	0.056	0.97 - 1.08	0.99 (0.96 - 1.02)	0.59

*The heterogeneity is given in terms of the 95% mid-range for practices. This is the range of rate ratios for the "true" step changes we expect to see across 95% of practices after accounting for patient sample size. This has been estimated using the standard deviation of the random slope for the step change (σ_{step}) combined with the fixed effect of the step change (β_{step}) as $e^{\beta_{step} \pm 1.96\sigma_{step}}$.

3.6 Prescriptions from general practice

The introduction of a 'telephone first' approach was not associated with any differences in cost or numbers of prescriptions except for the cost (but not the number of items prescribed) of prescriptions for iron deficient anaemia (tables M and N). There is no clinical reason for this observation, and as it is not reflected in the quantities prescribed, it is likely to be an incidental observation unrelated to the introduction of the 'telephone first' approach. We also found small changes in antibiotic prescriptions (table M) – a small non-significant 2.8% increase in numbers of antibiotics prescribed at transition (p=0.088) followed be a small (5%)

increase in the previous rate at which antibiotic prescriptions were declining (p=0.048). There was no change in the cost of antibiotic prescriptions (table N).

Table M Effects of a 'telephone first' approach on the number of drug items prescribed per practice per month[1]

Drug type	Step change at transition		Change in slope following transition *		95% reference range for step change transition
	Proportional change on the number of prescriptions	P	Time trend	P	
All	0.988 (0.944 ,1.034)	0.608	0.952 (0.886 ,1.024)	0.186	0.988 to 0.988
Asthma	0.994 (0.965 ,1.025)	0.714	0.972 (0.927 ,1.019)	0.242	0.994 to 0.994
Congestive heart failure	0.996 (0.964 ,1.030)	0.836	0.974 (0.924 ,1.026)	0.320	0.996 to 0.996
Angina	1.000 (0.970 ,1.031)	0.993	0.974 (0.929 ,1.021)	0.267	1.000 to 1.000
Iron-deficiency anemia	0.988 (0.961 ,1.015)	0.376	0.98 (0.941 ,1.022)	0.352	0.988 to 0.988
Hypertension	1.003 (0.976 ,1.032)	0.810	0.988 (0.945 ,1.032)	0.573	1.003 to 1.003
Antibacterial drugs	1.028 (0.996 ,1.062)	0.088	0.950 (0.903 ,1.000)	0.048	1.028 to 1.028
All ACSC drugs	0.989 (0.951 ,1.029)	0.590	0.964 (0.906 ,1.026)	0.247	0.989 to 0.989

** Compared with the trend in intervention practices pre-launch, adjusted for national trends. Change over 12 months post transition*

Table N Effects of the 'telephone first' approach on the cost of prescriptions per practice per month[2]

Drug type	Step change at transition		Change in slope following transition *		95% reference range for step change transition
	Proportional change on the number of prescriptions	P	Time trend	P	
All	0.989 (0.943 ,1.037)	0.653	0.992 (0.920 ,1.069)	0.833	0.989 to 0.989
Asthma	1.004 (0.970 ,1.040)	0.806	0.975 (0.923 ,1.029)	0.357	1.004 to 1.004
Congestive heart failure	0.981 (0.946 ,1.017)	0.289	1.031 (0.974 ,1.091)	0.294	0.981 to 0.981
Angina	1.003 (0.969 ,1.039)	0.845	1.026 (0.972 ,1.082)	0.356	1.026 to 0.972
Iron-deficiency anemia	0.976 (0.938 ,1.017)	0.252	1.091 (1.032 ,1.153)	0.002	0.955 to 0.998
Hypertension	1.001 (0.966 ,1.038)	0.947	1.01 (0.956 ,1.067)	0.729	1.001 to 1.001
Antibacterial drugs	1.031 (0.990 ,1.074)	0.136	0.982 (0.922 ,1.045)	0.562	1.031 to 1.031
All ACSC drugs	0.992 (0.951 ,1.035)	0.723	0.99 (0.927 ,1.059)	0.778	0.992 to 0.992

** Compared with the trend in intervention practices pre-launch, adjusted for national trends. Change over 12 months post transition*

[1] Drugs for chronic obstructive pulmonary disease are dropped since there's no observation in 2012, 2014 and 2015.

[2] Drugs for chronic obstructive pulmonary disease are dropped since there's no observation in 2012, 2014 and 2015.
