# Supplementary material for: Evaluation of telephone first approach to demand management in English general practice: observational study
Source: BMJ. 2017 Sep 28;358:j4197. doi: 10.1136/bmj.j4197 (PMC5615264; doi:10.1136/bmj.j4197)
Supplement: Supplementary file 4 — Appendix 4: Practice manager survey [file newj040067.ww4.pdf]

Appendix 4. Practice Manager Survey [posted as supplied by author]

Practice manager survey: methods

A survey was sent to the managers of all 101 practices that had been running the ‘telephone first’ approach for at least six months asking about their experience of the new approach and their perception of advantages and disadvantages for both staff and patients.

Practice manager survey: results

Of 42 respondents to the practice manager survey (response rate 41.6%), most respondents replied to all questions. 87.8% (36/41) reported advantages for GPs and practice staff and 78.1% (32/41) reported disadvantages. The most frequently reported advantages were GPs being able to manage their time better (9), the increase in available appointment slots (5) and less pressure on receptionists having to act as gatekeepers for appointments (4). The most frequently reported disadvantages were an increase in overall workload (11), increased demand from patients (9) and increased patient complaints (4). Likewise, practice managers identified both advantages and disadvantages for patients. The most commonly reported advantages were improved access (8), patients not having to take time off work for an appointment (7) and shorter waiting time for appointments (6), with the commonest reported disadvantages that the system was more challenging for some groups such as deaf or hearing impaired people or those with poor English (10), the lack of pre-bookable appointments (7) and patients not wishing to discuss their problem on the phone (7).
